# The Secreted Protease PrtA Controls Cell Growth, Biofilm Formation and Pathogenicity in *Xylella fastidiosa*

**DOI:** 10.1038/srep31098

**Published:** 2016-08-05

**Authors:** Hossein Gouran, Hyrum Gillespie, Rafael Nascimento, Sandeep Chakraborty, Paulo A. Zaini, Aaron Jacobson, Brett S. Phinney, David Dolan, Blythe P. Durbin-Johnson, Elena S. Antonova, Steven E. Lindow, Matthew S. Mellema, Luiz R. Goulart, Abhaya M. Dandekar

**Affiliations:** 1 Plant Sciences Department, University of California, Davis, CA, USA; 2 Institute of Genetics and Biochemistry, Federal University of Uberlândia, Brazil; 3 Proteomics Core, Genome Center, University of California, Davis, USA; 4 Public Health Sciences, University of California, Davis, USA; 5 Plant and Microbial Biology Department, University of Berkeley, Berkeley, CA, USA; 6 Surgical and Radiological Sciences, Vet Med, University of California, Davis, CA, USA; 7 Department of Medical Microbiology and Immunology, University of California, Davis, CA, USA

## Abstract

Pierce’s disease (PD) is a deadly disease of grapevines caused by the Gram-negative bacterium *Xylella fastidiosa*. Though disease symptoms were formerly attributed to bacteria blocking the plant xylem, this hypothesis is at best overly simplistic. Recently, we used a proteomic approach to characterize the secretome of *X. fastidiosa,* both *in vitro* and *in planta*, and identified LesA as one of the pathogenicity factors of *X. fastidiosa* in grapevines that leads to leaf scorching and chlorosis. Herein, we characterize another such factor encoded by PD0956, designated as an antivirulence secreted protease “PrtA” that displays a central role in controlling *in vitro* cell proliferation, length, motility, biofilm formation, and *in planta* virulence. The mutant in *X. fastidiosa* exhibited reduced cell length, hypermotility (and subsequent lack of biofilm formation) and hypervirulence in grapevines. These findings are supported by transcriptomic and proteomic analyses with corresponding plant infection data. Of particular interest, is the hypervirulent response in grapevines observed when *X. fastidiosa* is disrupted for production of PrtA, and that PD-model tobacco plants transformed to express PrtA exhibited decreased symptoms after infection by *X. fastidiosa*.

The bacterium *Xylella fastidiosa* is a versatile, xylem-limited, insect-transmitted, Gram-negative member of the *Xanthomonadaceae* that causes disease in many economically important crops around the world. Grape growing regions of California are endemic for strains of *X. fastidiosa* that cause Pierce’s disease (PD)^1^ and harbor many species of xylem sap-feeding sharpshooter insects that vector the pathogen from plant to plant, including the glassy-winged sharpshooter (GWSS)^2^. To avoid epidemics like those seen in the late 90’s, the wine, raisin and table grape-producing regions of the state have adopted very labor- and cost-intensive integrated management practices^2^. Recent research has focused on understanding the pathobiology of *X. fastidiosa* in grapevine, in hope that this understanding will allow a less costly, long-term defense against PD.

Subsequent studies on *X. fastidiosa* identified several virulence factors, some of which are secreted into the extracellular microenvironment^3^,^4^. Broadly, bacterial secreted factors play an important role in plant-pathogen interactions, potentially triggering pathogenicity^5^,^6^,^7^,^8^,^9^,^10^. Indeed, pathogenicity-related proteins secreted into the environment can facilitate the processes of infection and invasion^5^,^9^,^11^. Remarkably, expression of many such secreted proteins depends on the aggregation state of the microorganism, which exists either in a planktonic, motile condition or associated with other cells and an extracellular matrix within a biofilm^1^,^12^,^13^,^14^. While in planktonic form, *X. fastidiosa* expresses an array of proteins involved in cell wall and pit membrane degradation and uses Type IV pili (required for long-distance twitching movement) to colonize more vessels^1^,^15^. *X. fastidiosa* is considered more virulent when in a planktonic state, as mutant strains which form less biofilm induce early disease symptoms upon inoculation into grapevines^16^. However, this idea conflicts with prevailing thought about the overall mechanism of disease induction and progression in *X. fastidiosa*: it has been posited to occlude xylem sap pathways either by forming aggregates within exopolysaccharide-rich biofilms^17^,^18^ or by inducing callose and tylose formation^19^, thereby inducing water stress in plants and eventual vine death. Contrary to this hypothesis, disease symptoms are not completely replicated by inducing water stress and bacterial cells are often not found near areas of PD symptoms^20^, indicating that further research is necessary to fully understand this plant-pathogen interaction.

Our previous work showed that LesA (PD1703) may serve as a key secreted virulence protein capable of inducing scorching symptoms in grapevines^4^. Additional work has identified other secreted virulence factors including biofilm-promoting proteins, exoenzymes, and outer membrane vesicles^21^,^22^,^23^,^24^. As such, the water stress hypothesis provides at least an oversimplified view of disease induction, one that fails to describe the role and importance of *X. fastidiosa*’s secreted products. Here we describe the role of one of these, a secreted serine protease encoded by PD0956 in the PD-causing strain Temecula1.

## Results

### PD0956 is a secreted serine protease

**Figure 1:**
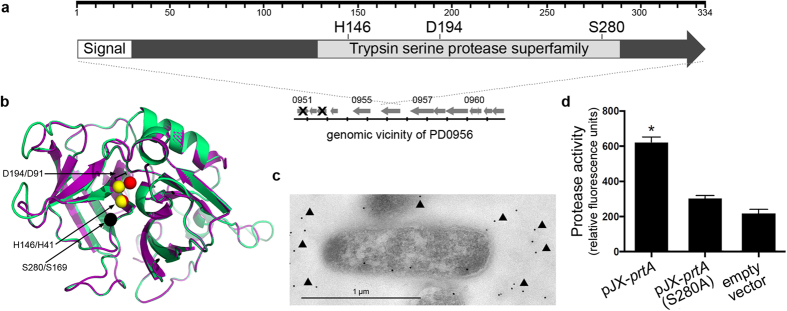
PD0956 functional characterization. (**a**) Schematic representation of the coding region of PD0956 including the 28 amino acid N-terminal signal peptide (MRAIKLNKLSLGLLGIFSLTLIPSLSIA), and the position of the predicted catalytic active residues (H146, D194, S280). (**b**) Superimposition of predicted structures for PD0956 (protein in green, catalytic residues in red) and an extracellular alkaline serine protease (PDB: 3CP7A, protein in purple, residues in yellow) showing the conserved catalytic triad. The serine residue overlaps completely (in black). Superimposition was carried out using MUSTANG. (**c**) Immunogold labeling of *X. fastidiosa* PD0956. Gold particles are associated with the bacterial secreted matrix (black arrowheads). (**d**) Protease activity for wild type PD0956 expressed in *E. coli* (pJX-*prtA*), an empty vector control, and the PD0956 functional mutant (pJX-*prtA*-S280A). The functional mutant contains a substitution in the predicted catalytic residue. Activity was quantified using fluorescein labeled casein as substrate. Average values and standard deviations of three independent experiments are plotted (*p-value < 0.05, two-tailed T-test).

**Figure 2:**
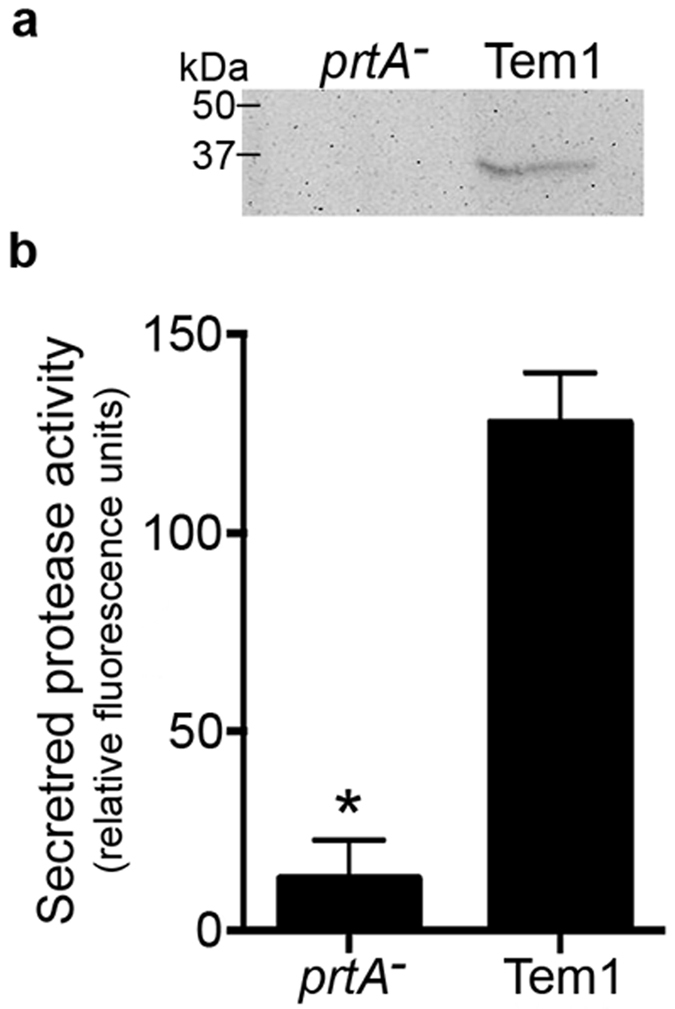
PD0956 is a serine protease expressed in *Xylella fastidiosa*. (**a**) Immunoblot detection of secreted PrtA. A cropped blot is shown in this figure and the whole blot image is shown in the Supplementary Information file. (**b**) Fluorescent protease activity assay (FTC-Casein) showing reduced activity in secreted extracts of *X. fastidiosa prtA* mutant vs wild type Temecula1. Average values and standard deviations of three independent experiments are plotted (*p-value < 0.05, two-tailed T-test).

A previous analysis of the *X. fastidiosa* secretome identified a putative uncharacterized protein (locus tag PD0956)^4^. Sequence analysis of this protein reveals a conserved trypsin-like peptidase domain, the presence of an N-terminal secretion signal peptide, and similarity to other putative extracellular serine proteases such as emb|CAN00053.1| (Fig. 1a). Furthermore, there is close superimposition of the three-dimensional structure from the *Xylella* protein onto an alkaline serine protease from *Clavibacter michiganensis* (Fig. 1b), particularly the catalytic triad within the active site. The protein immuno-localizes to the extracellular vicinity of isolated *X. fastidiosa* cells (Fig. 1c), confirming it to be a secreted product. The recombinant protein produced and secreted by *Escherichia coli* possesses serine protease activity, but not when the catalytic site is mutated (Fig. 1d). Finally, a *X. fastidiosa* insertion mutant fails to produce a detectable product using anti-PrtA in secreted extracts (Fig. 2a) drastically reducing serine protease activity in secreted extracts (Fig. 2b). As such, the *Xylella* protein PD0956 has been annotated “PrtA”, a secreted serine protease with confirmed functional activity and site of action.

### PrtA promotes large-scale changes of *X. fastidiosa* transcriptome and proteome

PD0956 encodes a previously uncharacterized protein of ~37 kDa detected in the secretome of *X. fastidiosa* culture supernatant^4^. Part of a bacteriophage remnant sequence (spanning PD0951 to PD0943, Supplementary Information Fig. S1), orthologs are present and highly conserved in about half of the *Xylella* spp. genomes sequenced to date (not shown). To understand its functional role, we disrupted its coding sequence (Supplementary Information Fig. S2) and analyzed the resulting phenotype, including transcriptome and secretome changes (Table 1 and Supplementary Information Tables S1 and S2). Disruption of PD0956 significantly changed expression of 684 protein-coding transcripts (32.3% of 2118 detected) and 92 secreted proteins (31.5% of 292 detected) in many diverse functional categories as detailed in the gene ontology (GO) analysis (Supplementary Table S3). In general terms, the GO analysis of the differentially expressed transcripts highlighted lipid A biosynthesis (GO:0009245), amino acid transport (GO:0006865), SOS response (GO:0009432), pathogenesis (GO:0009405), and protein turnover (GO:0030163, GO:0006412), suggesting an adaptation to intense cell proliferation and attenuation of microbial stress defenses. Specific genes/pathways are discussed in following sections according to their cellular and biochemical context within the mutant phenotype characterization. We considered a polar effect on downstream genes from the insertional mutation as PD0954 encodes an Xre-family transcriptional regulator, but both PD0955 and PD0954 remained transcriptionally active in the RNA-seq data of the *prtA* mutant. Moreover, PD0956 and the downstream coding sequences belong to independent transcriptional units according to the Prokaryotic Operon DB^25^ and DOOR 2.0 server^26^.

### PrtA affects biofilm formation, motility, and cell length

**Figure 3:**
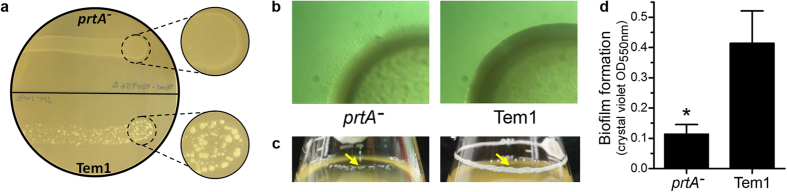
PrtA alters motility and biofilm formation. (**a**) Comparison of growth patterns of the *prtA* mutant with wild type *X. fastidiosa* on a PD3 agar plate, indicating differential cell aggregation. (**b**) Colony morphologies displaying increased twitching motility of *prtA* over wild type *X. fastidiosa* strain Temecula1 grown on PD3 for eight days. Notice the increased fringe zone formed by cells performing twitching motility on the colony periphery of *prtA* mutant cells. (**c**) Minimal biofilm formation by the *prtA* mutant in broth culture, unlike wild type *X. fastidiosa*. (**d**) Quantification of biofilm formation at the air-liquid interface using crystal violet staining after seven days growth in liquid PD3 at 95 rpm and 28 °C. Average values (n = 8) and standard deviations are plotted (*p-value < 0.001, two-tailed T-test).

Cultivation of the *X. fastidiosa prtA* mutant on agar plates provided an initial indication of altered cell aggregation and microcolony formation. Mutant cells formed a uniform streak of biomass instead of small aggregates and microcolonies as commonly seen using wild type Temecula1 (Fig. 3a). We hypothesized that an aggregation-deficient mutant would also exhibit hypermotility^27^, confirmed by a broader fringe zone at the colony periphery in the mutant (Fig. 3b) which indicates greater twitching-motility^4^. Increased motility was further implicated by inability to form biofilm in rotated culture broth (Fig. 3c,d). The *prtA* mutant strain grows predominantly in the planktonic bioform, with a distinct lack of biofilm formation compared to its parental strain. Transcriptome analysis of the mutant and wild type correlate with this data, as genes important for cell aggregation, adhesion and biofilm development were down-regulated in *X.fastidiosa prtA*^*−*^. These include *fimC* (PD0061) and *mrkD* (PD0058) involved in chaperone-usher pilus formation, the trimeric autotransporter adhesin *xadA3* (PD0824), fastidian gum synthesis *gumM* (PD1387), the carbamoyl-phosphate synthase operon *carAB* (PD0398 and PD0399), and neighboring genes (part of the same operon) *greA* (PD0400) and *rpfE* (PD0401). Interestingly, one *pspA* hemagglutinin-like paralog (PD0986) was down-regulated while two (PD2116 and PD2110) were up-regulated in the *prtA* mutant. *X. fastidiosa* hemagglutinins act as antivirulence factors, facilitating self-aggregation and attenuating pathogenicity by limiting host colonization capacity^28^.

The transcriptome and proteome data provide further insight into the increased motility of *X.fastidiosa prtA*^*−*^, with up-regulation of *pilA* (PD1924), *pilE* (PD0024), two homologs of *pilY1* (PD0023 and PD1611), *pilP* (PD1692), *pilS* (PD1929), and *fimT* (PD1735) when compared to wild type. These are involved in the assembly and function of type IV pili and associated with *X. fastidiosa* biofilm formation and pathogenicity^27^,^29^, with mutation of *pilY1* decreasing twitching motility in *X. fastidiosa*^30^. Moreover, the two-component system AlgZR encoded by PD1154 and PD1153 controls twitching motility in *Pseudomonas aeruginosa*^31^ and was up-regulated in our *prtA* mutant. Interestingly, the response regulator PD1386 showed intense induction in the *prtA* mutant, but is yet uncharacterized and its sensor partner is unknown.

**Figure 4:**
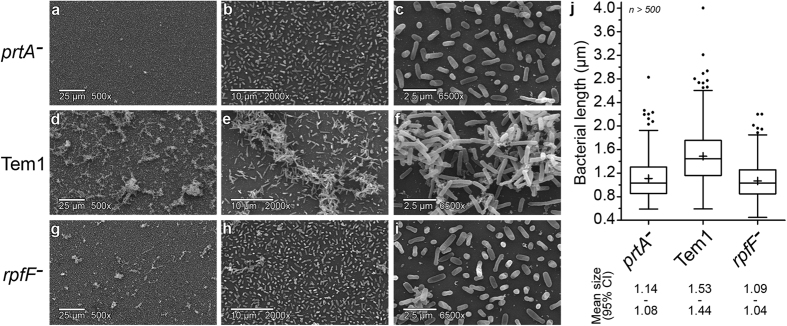
Reduced cell length as an indicator of increased motility and decreased biofilm formation. Scanning electron microscopy of *prtA*^−^ (**a**–**c**), wild type (**d**–**f**), and *rpfF*^−^ (**g**–**i**) cells showing differential microcolony formation and cell length with a minimum of 500 bacteria measured per strain. (**j**) Box plot showing distribution of cell lengths according to cell types. Size ranges within 95% confidence intervals (CI) are indicated below the graph.

Scanning electron microscopy (SEM) confirmed the predominant planktonic bioform of the *prtA* mutant (Fig. 4a–c), compared to wild type Temecula1 cells (Fig. 4d–f). This predominant planktonic behaviour is similar to that observed for biofilm-deficient *rpfF*^−^ cells (Fig. 4g–i). SEM also revealed a striking reduction in cell length compared to the parental wild type, with an attenuation of the rod-like shape. Again, wild type and *prtA* mutant transcriptome comparisons show significant differential regulation of the rod-shape determining genes *mreB* (PD0557), *mrdB* (PD0561), and *pbp* (PD1157, a beta lactamase-like gene essential for cell elongation). Misregulation of these genes can influence cell morphology in other rod-shaped bacteria^32^. Further, reduced cell length indicates rapidly dividing cells, which is supported by the increased transcription of cell division regulator *fic* (PD1006), septum formation and cell division genes *ftsH* (PD0070), *ftsJ* (PD0071), *nlpD* (PD1820), *ftsZ* (PD1861), peptidoglycan recycling *mltB* (PD1235) and *mviN* (PD1439), and a glycine cleavage system component *gcvH* (PD0148), important for glycine turnover to prevent inhibition of cell growth effect on peptidoglycan synthesis^33^. Finally, the biosynthesis of many cellular components was up-regulated, serving as another indicator of rapid cell division. These include cell wall synthesis *pgpB* (PD1955), *lpxK* (PD0362), *lpxA* (PD0323 and PD1769), *cdsA* (PD0329, down-regulated in elongated *Bacillus subtilis* cells^34^), *htrB* (PD0592), *ampE* (PD0825), *wbpL* (PD1452), isoprenoids *ispG* (PD1956), nucleotides *pyrE* (PD0122), *purA* (PD1627), *purE* (PD2036), amino acids *argH* (PD0295), *proB* (PD0296), *proA* (PD0297), *pheA* (PD0426), *aroK* (PD0582), *asd* (PD0608), *aspH* (PD0777), *trpDE* (PD0876 and PD0877), *serC* (PD1358), *leuB* (PD1397), cofactor recycling *cysG* (PD1840), *folP* (PD0068), *ptr1* (PD0677), *nadA* (PD0869), *grx* (PD1409), *ribF* (PD1438), *folB* (PD1642), terpenoid *dxr* (PD0328), phosphate uptake *oprO* (PD0270), ammonium uptake *amtB* (PD1024), sugar ABC transporter *malGFE* (PD1464-6), and energy metabolism *gpmA* (PD0898), also up-regulated in planktonic *Salmonella enterica*^35^. As SEM preparations may destroy three-dimensional structures during sample preparation, it is of particular interest that the planktonic lifestyle of a given strain of *X. fastidiosa* could be associated and identifiable through an analysis of bacterial length, as demonstrated here for both the *prtA* and *rpfF* mutants (Fig. 4j). We also have unpublished observations that the citrus-infecting *X. fastidiosa* strain Fb7 also displays these characteristics of shorter cell length, hypermotility and lack of biofilm formation (in preparation). This correlation should be further investigated with other *X. fastidiosa* strains and mutants, as it could potentially become a valuable parameter for virulence assessment of different isolates.

### Secreted PrtA limits bacterial proliferation and the quantity of extracellular DNA

**Figure 5:**
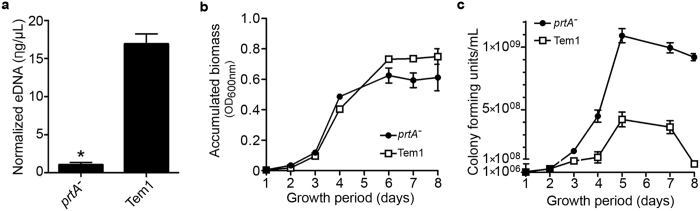
PrtA influences eDNA production and cell proliferation. (**a**) Quantification of extracellular DNA present on biofilm formed by *X. fastidiosa prtA*^*−*^ mutant and wild type after seven days in broth culture. Average values and standard deviations of three independent experiments are plotted (*p-value < 0.05, two-tailed T-test). (**b**) Accumulation of biomass in broth cultures measured by absorbance at 600 nm. (**c**) Evaluation of the number of colony-forming units (CFU) over eight days of culturing.

Extracellular DNA (eDNA) is a required macromolecule for biofilm formation^36^. Bacteria provide the required eDNA using different mechanisms including autolysis, which involves extracellular enzymes^37^,^38^. As PrtA showed to be important for biofilm formation, we hypothesized that it would be involved in eDNA formation and therefore that *X. fastidiosa prtA* mutant cultures would contain less eDNA than biofilm made by the wild type, as verified in equivalent biomasses (Fig. 5a). Moreover, despite similar overall turbidity of the culture broth (Fig. 5b), the *prtA* mutant strain has more living cells in culture than its parent strain (Fig. 5c). Interestingly, this difference was identified by counting plated colony forming units (CFU), but not detectable by comparing direct absorbance (medium turbidity) values (Fig. 5b).

Transcriptomic and proteomic data comparing the mutant and wild type supports PrtA’s role in eDNA formation and control of cell proliferation. Cell lysis transcripts *secE* (PD2007), *secG* (PD0246), *tatB* (PD1579), and the colicin V-like *cvaC* (PD0215-6) and secretion partner *cvaA* (PD0496) are all significantly down-regulated in this mutant. Colicins are known agents of cell lysis and eDNA formation, reviewed in ref. 39. On the other hand, up-regulation of cell division and biosynthesis transcripts, plus increased concentrations of ribosome subunits (nine out of 10), transfer RNAs (43 out of 49), tRNA maturation *queA* (PD0562), elongation factor *efp* (PD1253), endoribunuclease *ybeY* (PD1781), and chaperone *dnaJ* (PD1279) to assist in protein folding, are all indicative of the increased cell division rate in *X. fastidiosa prtA*^*−*^.

### Despite increased cell proliferation, DSF production and outer membrane vesicle secretion are unaffected by PrtA inactivation

**Figure 6:**
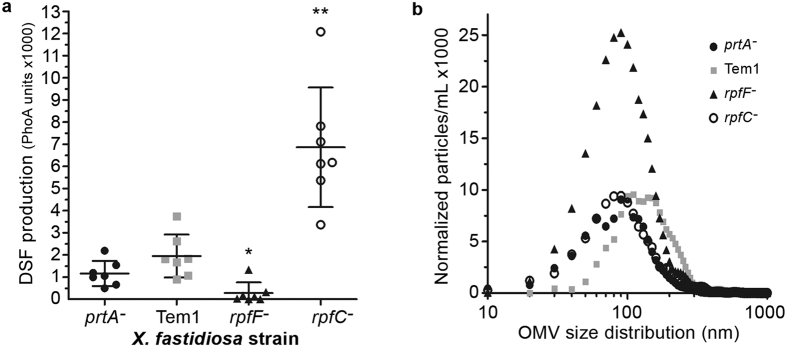
DSF and OMV production are not affected by PrtA. (**a**) *Xylella fastidiosa* biosensor strain showing significant increases (*rpfC*^*−*^ control) and decreases (*rpfF*^*−*^ control) in DSF relative to wild type Temecula1, but no significant changes in the *prtA* mutant. Response to DSF is measured via alkaline phosphatase activity of the *hxfA’*::*phoA* reporter gene fusion. All independent measurements were plotted (n = 7) and averages plus standard deviations are indicated. Groups significantly different to the wild type are indicated (*p-value < 0.05, **p-value < 0.01, ANOVA). (**b**) Quantification of number and size of outer membrane vesicles by nanoparticle tracking analysis isolated from wild type *X. fastidiosa* and mutant *prtA*, *rpfC*, and *rpfF* strains after five days growth in PD3 broth cultures. Plotted values are averages of 5 replicates after combining 5 independent cultures for each strain. This was repeated three times independently.

Recent work performed by the Lindow laboratory showed an interesting connection between diffusible signal factor (DSF) and vesicle production by way of the RpfF/RpfC quorum sensing system. The *X. fastidiosa rpfF* mutant is deficient in DSF production, produces extreme numbers of outer membrane vesicles (OMVs), lives predominantly in the planktonic bioform, and is hypervirulent^24^. Since the *prtA* mutant resembled the *rpfF* mutant in terms of shortened cell length, hypermotility, and lack of biofilm formation, we considered a connection with DSF signaling. As the *prtA* mutant is highly planktonic, we investigated whether DSF concentrations were reduced as reported in the *rpfF* mutant using a *X. fastidiosa*-based DSF-biosensor harboring a *hxfA’*::*phoA* reporter gene fusion^40^. Different from *rpfF*^*−*^ though, the *prtA* mutant had comparable DSF concentrations to the WT (Fig. 6a). This confirms previous work that DSF concentrations are determined by the number of bacteria present or “quorum sensing”^40^, but it also indicates a separation between the planktonic hypervirulent lifestyle and decreased DSF production as observed in the *rpfF* mutant^41^. As such, a decrease in DSF is correlated with increased pathogenicity, but the converse is not true; increased pathogenicity is not necessarily related to decreased DSF production.

Another interesting contrast to *rpfF*^*−*^ concerns membrane vesicle production. Nanoparticle tracking analysis showed equivalent secretion of outer membrane vesicles (OMVs) by *X. fastidiosa prtA* and wild type Temecula1 (Fig. 6b), while the *rpfF* mutant was confirmed to produce copious amounts of OMVs. This reinforces the hypothesis that RpfF and therefore DSF and quorum sensing are strongly involved in regulating vesicle formation, but also indicates that PrtA suppresses pathogenicity in *X. fastidiosa* via a separate regulatory pathway.

### PrtA is an antivirulence factor in Pierce’s disease

**Figure 7:**
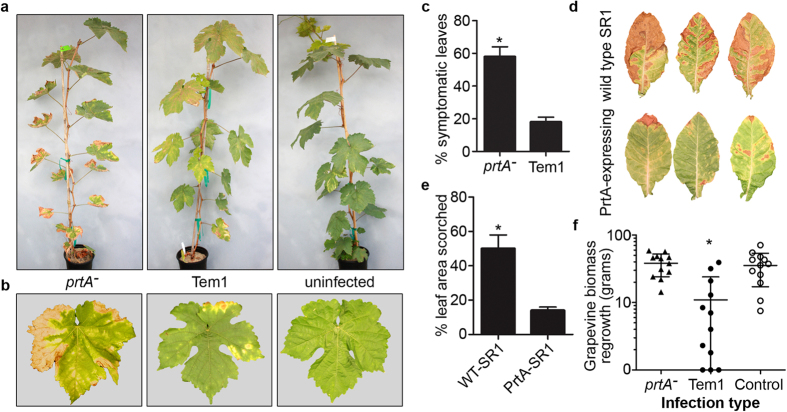
PrtA is an antivirulence factor in Pierce’s disease. (**a**) Plants infected with wild type Temecula1 and *prtA* mutant strain at eight weeks post-inoculation and (**b**) detached leaves harvested eight nodes above point of inoculation. (**c**) Quantification of symptoms averaged from 25 plants/strain infected with *X fastidiosa prtA* or wild type Tem1 control. Data is shown as the difference from uninfected control. Plotted values are averages and standard deviations from all plants tested (*p-value < 0.05, two-tailed T-test). (**d**) Comparison of leaf scorching symptoms in *X. fastidiosa*-infected wild type SR1 tobacco and SR1 expressing recombinant PrtA, after 11 weeks infection. (**e**) Quantification of leaf scorching area in wild type and PrtA-expressing SR1 tobacco. Plotted values are averages and standard deviations from all symptomatic leaves (n = 10 for WT SR1 tobacco and n = 10 for PrtA-expressing tobacco plants) from six plants of each genotype tested (*p-value < 0.05, two-tailed T-test). (**f**) Accumulated biomass of new vine buds grown for four weeks and then pruned. Plotted values are averages and standard deviations from total new biomass from a random subset (n = 12) of plants (*p-value < 0.05, ANOVA).

As reduced biofilm formation and increased twitching motility are associated with hypervirulence in *rpfF*^*−*^, these *in vitro* phenotypes of *prtA* mutant prompted us to verify its virulence to grapevines. Conventionally for this pathosystem it was believed that disease symptoms are a direct result of *X. fastidiosa* biofilm blocking the xylem. By this simplistic explanation, biofilm-deficient *X. fastidiosa prtA*^*−*^ would be less virulent, or at least display delayed onset of symptoms. However, like the planktonic *rpfF* mutant, the planktonic *prtA* mutant was also hypervirulent in grapevines, and the *prtA* mutant shows early onset of disease. Thompson Seedless grapevines infected with the *prtA* mutant strain showed severe PD symptoms significantly earlier than plants infected with the WT strain. The onset of PD scorching symptoms began six to eight weeks post-infection in *X. fastidiosa prtA*, but eight to ten weeks post-infection in the WT controls. This is seen in a visual comparison of PD eight weeks post-infection (Fig. 7a,b) and after quantification of disease symptoms at 10 weeks post-infection (Fig. 7c). *X. fastidiosa prtA* mutant-infected plants clearly display significantly increased disease symptoms when compared to control plants.

The quicker disease onset detected for the *rpfF* and *prtA* mutants not only correlated with the planktonic lifestyle and increased cell proliferation as shown previously, but also to changes in transcript levels associated with virulence mechanisms such as: (1) Increased expression of *xpsE* and *xpsF* (PD0732-3). These are orthologs of *gspE* and *gspF* in *Pectobacterium carotovorum,* and code for components of a Type II Secretion System (T2SS) cytoplasmic ATPase (GspE) and inner-membrane platform protein (GspF)^42^. (2) Increased *pefK* (PD0738), encoding another component of the T2SS. These secretion systems are pivotal for virulence of many plant pathogens and required for secretion of extracellular enzymes such as lipases, proteases, cell wall-degrading enzymes and factors responsible for host-pathogen interaction^11^,^43^,^44^. (3) Up-regulation of *cbsA* (PD0529), a cell wall-degrading enzyme. (4) Up-regulation of the PD1341-3 cluster encoding the virulence-associated protein VapI and the toxin/anti-toxin pair HicAB.

To further confirm the antivirulence activity of PrtA, the coding sequence was expressed in SR1 tobacco plants. A plant *prtA* transgene was created by removing the 28-amino acid bacterial signal sequence (Fig. S2c) and replacing it with a plant-derived signal peptide sequence (Ramy3D) previously used to secrete proteins to the plant apoplast. A binary vector, pDG14.01, was constructed to express the signal peptide-fused PrtA protein-coding region using CaMV35S 5′ and 3′ regulatory sequences and used to transform SR1 tobacco. Two transgenic SR1 tobacco lines expressing PrtA were challenged with *X. fastidiosa* and displayed increased protection against *X. fastidiosa* as quantified by leaf scorching area (Fig. 7d,e). These data further support an antivirulence role for PrtA.

### Loss of PrtA function decreased stunting symptoms in grapevines infected with *X. fastidiosa*

At 18 weeks post-infection, grapevines were cut and allowed to regrow for one month after which the new tissue was removed and weighed. Interestingly, vines infected with *X. fastidiosa prtA*^*−*^ lack the stunting phenotype associated with PD^45^, characterized by a reduction in regrowth of new buds after pruning of *X. fastidiosa*-infected vines (Fig. 7e). The transcriptome data helps us interpret this: down-regulation of stress tolerance genes would reduce the bacterial resistance to plant’s defenses activated upon wounding from pruning. This altered gene expression coupled to the absence of biofilm formation exposes *prtA* mutant cells to more environmental hazards that possibly limit pathogen persistence and allows for normal biomass formation in new buds. One of the main plant defenses against wounding and invading pathogens is generation of reactive oxygen species (ROS) that serve in microbial clearing, host cell wall strengthening and plant signaling^46^. On the other hand, bacteria can handle stressors such as ROS through the use of the SOS response; a response which delays cell division and repairs damaged DNA among other traits^47^. Genes within this circuitry are usually kept silent by the LexA repressor (PD0092 in *X. fastidiosa*) and de-repressed after autolysis of LexA during stress^47^. Organisms have developed adaptations in the SOS response, which can include biofilm formation such as in *Listeria monocytogenes*^48^. This LexA repressor is up-regulated in *prtA*^*−*^. Further, *priA* (PD2047), encoding a DNA replication protein which induces SOS response when inactivated^49^, was up-regulated in our data. Silencing of the SOS response is further suggested by down-regulation of regulon members *dinD* (also known as *pcsA*, PD1061), *recJ* (PD0402), and *recF* (PD0003) in the *prtA* mutant. Prevention of cell division by inactivation of FtsZ has also been documented as part of the SOS regulon that can lead to cell elongation while stress persists^50^. As mentioned previously, *ftsZ* and other CDS involved with cell division were down-regulated in *prtA*^*−*^. Taken together these data argue in favor of SOS response silencing in the mutant, possibly reducing its resistance to environmental stress. The suggested increased sensitivity of the *prtA* mutant to environmental stress is further supported by the down-regulation of *algU* (PD1284), for which inactivation also impairs biofilm formation in *Burkholderia pseudomallei*^51^ and reduces stress tolerance in *X. fastidiosa*^52^.

## Discussion

Bacterial pathogenesis has been commonly enhanced by secretion and action of virulence factors. More recently, a new class of ‘antivirulence’ genes has been identified in different pathogens, and disruption of antivirulence factors results in hypervirulent phenotypes^53^. We describe here the functional characterization of the secreted protease PrtA, which functions as an antivirulence factor in *X. fastidiosa*. Disruption of *prtA* (PD0956) in *X. fastidosa* increased virulence in inoculated grapevines, as predicted from its *in vitro* phenotype of faster proliferation and increased motility. The observed phenotype could also be anticipated from the transcriptomic and proteomic data, which indicated up-regulation of genes involved in cell division, biosynthesis of amino acids and proteins, nucleotides, cell wall components, many secondary metabolites, and type IV pilus which is responsible for twitching motility (the only form of motility performed by *Xylella spp.*). The omics data indicates down-regulation of fastidian gum production and adhesion molecules important for biofilm development. These data corroborate the *in vitro* phenotypes displayed by the *prtA* mutant, but there were some incongruities between transcriptome and proteome data. For example, the most up-regulated protein in the *prtA* mutant secretome was the argininosuccinate synthase encoded by *argG* (PD0291), yet no significant changes in its transcription were detected. This protein is involved in biosynthesis of arginine and was previously characterized as a virulence factor in *Xanthomonas oryzae pv. oryzae*^54^, but would not have been selected based solely on transcriptome data. This highlights both the usefulness and associated challenges of integrating multiple omics data. In general terms, the transcriptome data correlated better with the observed phenotypes *in vitro* and *in planta*, perhaps due to its true “whole genome” screening characteristic and higher sensitivity of less-abundant transcripts.

Remarkably, disruption of *prtA* greatly increased cell division, and thus a faster generation time may be responsible for the decreased average bacterial length seen for this and also for the planktonic and hypervirulent *rpfF* mutants. Care must be taken when working with multiple strains or lineages of *X. fastidiosa* since absorbance at 600 nm as a measure of cell density may not distinguish among strains of varying viability or length, nor distinguish between cells and other secreted biomaterials such as EPS and OMVs that also contribute to turbidity. Our data shows that normalizing bacterial suspensions of wild type versus *prtA*^*−*^ or *rpfF*^*−*^ cells by absorbance values would underestimate the number of viable cells in the mutant cell suspensions if calibration values from wild type growth curves are used. At least a set of calibration curves of OD vs. CFU should be performed with each cell lineage to exclude cell length biases in OD readings.

Reduced biofilm formation is a striking phenotype of the *prtA* mutant that can have direct implication in its survival against host defenses. Bacterial biofilms are structured sessile communities held together by a self-producing matrix composed of macromolecules like exopolysaccharide, proteins and extracellular DNA^55^. Formation of these immobile communities and their inherent resistance to antibiotics and the host immune system are key elements of their ability to persist and establish chronic infection^56^. In *Xylella* spp., biofilms not only protect cells from environmental hazards but also constitute a necessary “sticky” bioform for efficient vector acquisition and transmission^1^,^57^. Thus despite being hypervirulent in terms of disease progression, the reduction of biofilm formation as seen *in vitro* possibly may cause decreased vector acquisition and transmission of this bacterium between plants, such as seen previously in the hypervirulent yet planktonic *rpfF* mutant^57^. Increased acquisition and transmission would serve as a strong selective pressure favoring a phenotype with “reduced virulence but increased survival and transmission efficiency” as seen for the wild type harboring PrtA function. Social behaviors which depend on cell-cell signaling have been extensively studied in *Xanthomonas* and *Xylella* spp., revealing that local cyclic di-GMP pools can regulate specific protein-protein interactions and virulence functions independently, such as motility, expression of extracellular enzymes and biofilm formation^3^,^58^,^59^. Interestingly, extracellular protease expression is among the traits controlled by the DSF quorum sensing mechanism in these organisms. Indeed the *prtA* mutant resembles the *rpfF* mutant (DSF synthase) not only in cell behaviour and morphology (shorter rods) but also in modulation of transcriptome towards motility and inhibition of biofilm formation^41^. As we show DSF accumulation does not differ greatly from wild type levels, it may be that local cyclic di-GMP homeostasis is being disrupted indirectly by the degradation of other enzymes involved in its turnover. This, in turn, could account for the persistence of the *prtA* mutant in the exploratory bioform as opposed to entering the aggregative biofilm-forming phase.

Extracellular DNA (eDNA) is an abundant component of the bacterial biofilm matrix^36^,^55^ and is provided by various bacterial mechanisms. These include secreted factors, such as autolysins and proteases, to facilitate the lysis of bacterial cells and yield eDNA for biofilm structure^38^. Interestingly, *prtA* mutants produce substantially less eDNA *in vitro*, possibly as a result of faster cell proliferation and/or reduction of cell lysis when compared to the wild type. Moreover, down-regulation of biofilm components and up-regulation of motility factors may prevent the *prtA* mutant cells from reaching a biofilm development point requiring significant amounts of structuring eDNA. This may also contribute to the lack of grapevine stunting upon pruning, as bacteria within biofilms are expected to be more resistant to environmental pressures, such as additional changes in the host defense response after pruning^60^. Increased sensitivity to environmental pressures may also exist as a result of the marked down-regulation of stress-related genes such as the SOS response. Cell proliferation and enrichment for stress-induced genes (and accompanying behaviors such as energy production) are generally considered antagonistic behaviors and reflect cell resource management and strategy^61^. Aiming to separate these two processes, a study with *S. cerevisiae* found that stress-induced genes were regulated by their environment and not dependent on growth rate. If true in *X. fastidiosa*, and with PrtA acting as a secreted protease facilitating autolysis, this suggests that the down-regulation of stress-induced genes in *X. fastidiosa prtA* is not a consequence of accelerated growth. Instead, down-regulation would result from the removal of a stressor in the environment, thus permitting increased growth. Regardless, these findings have interesting potential application for *X. fastidiosa* control, as pruning strategies combined with strategies to increase the planktonic bioform might lessen *X. fastidiosa* bacterial titer *in planta*, hopefully below a transmission-effective threshold. So even though the infected plant would still be condemned, disease transmission could possibly be reduced.

Mutant *prtA* is both hypervirulent and planktonic, but *in vitro* produces similar concentrations of DSF to wild type Temecula1. Although DSF is important in regulating the planktonic to biofilm switch, there are mechanisms contributing to the planktonic bioform that supersede those that control DSF production and thus the two are not integrally connected. Further, though hypervirulent, the *X. fastidiosa prtA* mutant *in vitro* does not experience a corresponding extreme increase in vesicle production like the hypervirulent and planktonic quorum sensing mutant *X. fastidiosa rpfF*^24^. This suggests that if vesicles are involved in the disease process, the quantity of OMVs in *X. fastidiosa prtA* is likely not as important to virulence as other vesicle characteristics, such as the cargo they contain. Analysis of OMVs cargo may shed further light on the complex mechanism of PD induction. This further implies that vesicle up-regulation may not be a required virulence mechanism in *X. fastidiosa* and that RpfF and thus DSF plays a more direct role in vesicle regulation.

In conclusion, PrtA is a secreted protease in *X. fastidiosa* that influences many traits both *in vitro* and *in planta*. Based on *in vitro* experiments, disruption of *prtA* ultimately leads cells to confinement in an exploratory single-celled bioform capable of rapid cell division. Though hypervirulent in controlled inoculations in terms of disease onset and progression, the mutant showed decreased symptoms of bud regrowth stunting upon infection and subsequent pruning. The *X. fastidiosa prtA* mutant suppresses the SOS response, appearing to confer higher sensitivity to environmental stresses as shown by plant regrowth after pruning. Whether SOS suppression is a consequence of faster cell division or part of a specific regulatory mechanism remains unknown. Curiously, this antivirulence gene confers a balance between reduced virulence and increased tolerance to host defenses. As such, the identification of the precise targets of PrtA may provide useful insight on its role in this process(es). The *X. fastidiosa* Temecula1 genome has two other protein coding sequences with low similarity to PD0956 that also encode putative extracellular serine proteases, PD0955 and PD0657 (e-values 8e-11 and 7e-10, respectively). The first is within the same phage remnant as PD0956, while the latter is not within or near phage sequences. Given the broad effects of PD0956 inactivation on cell morphology and behaviour, and the widespread of phage sequences in available genomes of *Xylella* strains, the relationship of these other putative proteases to bacterial survival and plant disease is worth investigating.

## Materials and Methods

### *In silico* protein analysis

A BLAST search of PrtA (Accession id: WP 011097867.1) revealed a putative serine protease (Accession id: WP 011931294) from *Clavibacter michiganensis* as the match with greatest identity to PrtA. Thereafter, BLAST filtered only by PDB structures was performed; this revealed a secreted alkaline serine protease (PDB:3CP7), from a halophilic microbe in the *Nesterenkonia abyssinica* group as the structure with greatest similarity to PrtA. Next, the structure of PrtA was modeled using PDB:3CP7 as a template for SWISS-MODEL (PrtA.pdb in Dataset). Finally, PrtA’s catalytic triad was identified by querying the modeled structure using PDB:3CP7 as a motif for CLASP^62^. These proteins were superimposed using MUSTANG with PrtA’s signal peptide predicted using SignalP 4.1 and removed (Fig. 1).

### Cloning PrtA in an *E. coli* expression vector

The sequence of PrtA (PD0956) and PrtA-S280A was codon-optimized for expression in *E. coli*, chemically synthesized, and then cloned into the pJexpress-401 expression vector (DNA2.0; Menlo Park, CA). For pJX-*prtA*-S280A, serine residue S280, which is part of the catalytic triad, was substituted with alanine to create a functional mutation.

### Isolation of the *prtA* mutant strain

Complete method and demonstration of disruption of PD0956 (PrtA) is available as Supplementary Information.

### Protease activity assay

PrtA activity was measured using a PierceTM Fluorescent Protease Assay Kit (Thermo) using fluorescein-labeled casein that fluoresces in the presence of protease activity. *X. fastidiosa* wild type Temecula1 and *prtA*^*−*^ protease activities were quantified using 25 ug total secreted protein pooled from three 10 mL broth cultures as suggested by the manufacturer. The fluorescent intensity was measured (485/538 nm excitation/emission maxima) for three replicas of each genotype after incubation for one hour at room temperature using the fluorometre SpectraMax M2 (Molecular Devices). Significantly different results (p < 0.05 two-tailed T-test) are indicated with an asterisk.

### Western-blot analysis

Synthetic peptides corresponding to structural epitopes of PrtA from *X. fastidiosa* were used to generate the polyclonal antibody (GenScript, NJ). Complete method of Western blot procedure is described in the Supplementary Information file. Whole image of blot is shown in Supplementary Fig. S3.

### Immunogold electron microscopy

Immunogold Electron Microscopy (IEM) was performed using fresh cultures of *X. fastidiosa* cells and the filamentous network secreted in the culture supernatant. These were fixed with 4% paraformaldehyde in 1 M Sorensen’s phosphate buffer (pH 7.4) and then embedded in LR White resin as described^63^. Ultra-thin sections were cut and placed onto coated grids (200 mesh; treated with glow-discharge), followed by blocking with 1% fish gelatin for 30 min. Grids were blotted with anti-PrtA (1:500) antibody for 1 h at RT and then washed with PBS. The primary antibody was detected using anti-rabbit (1:50) antibody coupled to 10 nm gold particles. Unbound conjugate was removed by a step-wise sequence of washing with PBS. The preparation was negatively stained with 1% ammonium molybdate, air-dried, and visualized using a Philips CM120 (FEI/Philips Inc.) electron microscope at 80 KV.

### Bacterial viability and growth

Eighty microlitres OD_600 nm_ = 0.11 of wild type Temecula1 and *prtA* mutant *X. fastidiosa* strains were inoculated into 10 mL aliquots of PD3 broth in 50 mL conical tubes and grown with shaking at 150 rpm in a 28 °C incubator. Cultures were allowed to grow for eight days with periodic plating of strains on five PD3 agar plates per strain per time point. Plates were incubated at 28 °C for ~ two weeks before counting the colony forming units. This experiment was repeated independently at individual time points with similar results. OD_600 nm_ was measured every day and growth curves show the average of three independent cultures with standard deviation.

### eDNA extraction and quantification

Extracellular DNA was extracted as previously described^64^ using six independent 50 mL cultures of *X. fastidiosa*. Cells were grown in PD3 liquid media at 28 °C in a rotary shaker for six days. Extracted eDNA was quantified using Qubit 2.0 (Thermo Fisher Scientific, NY).

### Crystal violet biofilm quantification assay

Individual wells in 12-well plates containing three mililitres fresh PD3 per well were inoculated with 24 μL blank PD3 or OD_600 nm_ = 0.1 *X. fastidiosa* wild type Temecula1 or *prtA*^*−*^ suspensions, incubated at 28 °C, and grown at 95 rpm for seven days. The biofilm of each culture (n = 8) was quantified using crystal violet staining as previously described^65^.

### Twitching motility assay

*X. fastidiosa* wild type Temecula1 and *prtA*^*−*^ were assayed for twitching motility using an existing protocol substituting PD3 for modified PW with BSA^27^. Briefly, five microlitres bacterial suspensions in liquid medium at OD_600 nm_ = 0.1 were spotted onto 0.6% PD3 agar plates, allowed to dry five min in a laminar flow cabinet, and incubated at 28 °C for eight days. Colonies were transferred onto PW 0.6% agar plates with a sterile toothpick and incubated at 28 °C for three days; after incubation, colony morphology was examined using a dissecting microscope to visualize the twitching motility zone at the fringe of the colonies.

### SEM preparations and bacterial length measurements

Eighty microlitres of a OD_600 nm_ = 0.1 *X. fastidiosa* wild type Temecula1 and mutant strains *prtA*^*−*^ and *rpfF*^*−*^ were inoculated into 10 mL aliquots of PD3^66^ broth in 50 mL conical tubes and grown with shaking at 150 rpm in a 28 °C incubator. Bacteria were sampled six days after inoculation without centrifugation, incubated in modified Karnovsky’s fixative for one hour at room temperature and subsequently stored at 4 °C. Karnovsky’s fixative consists of 2.0% paraformaldehyde and 2.5% glutaraldehyde in 0.1 M Sorensen’s phosphate buffer at pH 7.3. Samples were prepared with the help of Patricia Kysar from the Electron Microscopy Lab at the University of California Davis. Samples were viewed on an FEI XL-30 TMP Scanning Electron Microscope (FEI Company, OR) and images acquired by Hyrum Gillespie. Finally, the lengths of ~500 bacteria/strain were measured using SEM pictures taken at 6500× magnification and ImageJ. Data was graphed as the percent total bacteria at each length. Previous independent SEM preparations of *X. fastidiosa* strains displayed similar phenotypic differences in clumping and length.

### DSF quantification

*X. fastidiosa* strains were grown on ~550 mL 1.5% agar PD3 plates (22 plates). Each plate was spotted 25 times with 5 μL *X. fastidiosa* OD_600 nm_ = 0.1 and grown for two weeks without antibiotic. Eleven plates of each strain were cut into five mm^3^ cubes and incubated with an equal volume of ethyl acetate for two hours with shaking every 30 min. Ethyl acetate and agar were separated using cheesecloth and concentrated by evaporation (in-vacuo) using a Rotavapor R evaporator (Buchi, Switzerland). DSF-containing residues were resuspended in three mL methanol and quantified. DSF was quantified with help from Dr. Elena Antonova from the laboratory of Dr. Steven Lindow in Berkeley CA according to a previously published protocol using a rpfF*-XfHA-biosensor developed at their lab^40^,^67^. All extracts were stored at −20 °C. Note: DSF was also extracted from strains grown in liquid cultures with similar results.

### Outer membrane vesicle isolation and quantification

Outer membrane vesicles (OMV) were isolated using a previously reported protocol^22^. Quantification of vesicles was performed with the help of Dr. Matt Mellema, UC Davis using a Nanosight LM10HS in which a true medium blank and FM 1–43 dye from Molecular Probes were used to distinguish OMV from background particles. FM dyes are lipophilic styryl compounds used in previous studies of vesicles and plasma membranes. Note: We suggest rinsing all ultracentrifuge and storage tubes with 0.1 μm filtered ddH_2_0 to reduce the particle background.

### Grapevine growth, infection, and quantification

Grapevines (*Vitis vinifera* L. cv. ‘Thompson Seedless’) were propagated clonally, greenhouse-grown in pots, and pruned to maintain one leaf per node and heights of one metre. Ten-week-old grapevines were infected at 8–12 cm above soil level using a pin with 10 μL PD3 medium at 2 × 10^8 ^cells/mL of either *X. fastidiosa prtA* mutant, wild type Temecula1, or PD3 medium (mock inoculation) as described^68^. This process was repeated the following day with independently grown cultures to ensure infection. Plants were placed in the greenhouse in a randomized block design and maintained for 10 weeks, at which time PD-like symptoms were quantified. Leaves with any scorching were defined as “symptomatic.” Data was taken from 76 plants with a minimum of 25 plants for each treatment and presented as the percentage of symptomatic leaves above the inoculation point. Missing leaves were excluded from this analysis, but a separate analysis counting missing leaves as “symptomatic” caused no significant changes in symptomatic percentages.

### Grapevine Regrowth

At 18 weeks post-infection, grapevines infected with *X. fastidiosa prtA*^*−*^ and Temecula1 were completely scorched. Subsets of 12 vines of each inoculation type, including healthy mock-infected controls, were subsequently cut off below the infection point down to two inches (to the wood cutting of initial propagation) and then allowed to regrow for four weeks, at which time all new tissue was removed and weighed.

### Expression of recombinant PrtA in SR1 tobacco

Following the addition of the rice alpha amylase (RAmy 3D, GenBank:M59351) secretion signal peptide at the N-terminal and Flag detection and purification tag (Sigma) at the C-terminal, the coding sequence for PrtA was synthesized after codon optimization for plant expression (GenScript, NJ). Using epicenter Infusion Recombination cloning (Madison, WI), the PrtA sequence was cloned into our binary vector pDU92.3103 such that the coding region was downstream of the cauliflower mosaic virus (CaMV) promoter and upstream of the CaMV terminator. This resulted in binary vector pDG14.01 (Supplementary Information Fig. S2). The constructed binary vector was provided to the Ralph M. Parsons Plant Transformation facility at the University of California, Davis. Ten independent transgenic SR1 tobacco lines for pDG14.01 were obtained and confirmed by PCR analysis of their transgenic DNA.

### SR1 tobacco infection and symptom quantification

Tobacco plants were grown in the greenhouse and infected as described^68^. Briefly, for each of two transgenic lines and wild type control, 12 plants were obtained and six were infected with *X. fastidiosa* culture while six were mock-infected with PD3 medium. For each plant, three leaves were infected through petioles with 2.14 × 10^6^ *X. fastidiosa* cells. At 11 weeks post-infection, infected leaves (n = 10 for WT SR1 tobacco and n = 10 for PrtA-expressing tobacco plants) were scored for percentage area scorched and pictures were taken.

### Protein extraction and proteomic data analysis

Complete method is available as Supplementary Information. Briefly six culture replicates of 50 mL were grown in PD3 media for seven days (120 rpm at 28 °C) and cells were separated by centrifugation and total protein from supernatant isolated and tryptic digested for mass spectra collection with an Orbitrap Q Exactive Plus mass spectrometre (Thermo Fisher Scientific, CA). Scaffold Q + (version Scaffold_4.4.0, Proteome Software Inc., Portland, OR) was used to analyze peptide and protein identifications. P-values for the strain effect were FDR-adjusted. Analyses were conducted using the R, version 3.1.0 (http://www.R-project.org/).

### RNA extraction, library preparation and transcriptomic data analysis

Complete method is available as Supplementary Information. Briefly RNA from six bacterial cultures was purified and depleted for ribosomal RNA. Strand-specific RNA-seq libraries were generated and sequenced on one lane of an Illumina HighSeq 2500 (Illumina, CA). Enrichment of gene ontology terms of differentially expressed transcripts was performed with BayGO^69^ using 500 simulations and a P*-*value cutoff of 0.05.

## Additional Information

**How to cite this article**: Gouran, H. *et al.* A Secreted Protease PrtA Controls Cell Growth, Biofilm Formation and Pathogenicity in *Xylella fastidiosa. Sci. Rep.* **6**, 31098; doi: 10.1038/srep31098 (2016).

## Supplementary Material

Supplementary Information

## Figures and Tables

**Table 1:** Summary of differentially expressed transcripts and secreted proteins in the Xylella fastidiosa PD0956 mutant.

Analyte	Up-regulated	Down-regulated	Detected^a^
CDS^b^ with functional annotation	276	222	1120
CDS without functional annotation	112	74	951
Total CDS	388	296	2071/2076
tRNA	43	0	47/49
Secreted proteins with known function	22	58	252
Secreted proteins without known function	2	10	40
Total proteins	24	68	292

^a^Relative to total counts in the Temecula1 genome.

^b^Protein coding sequences.
