# Supplementary material for: The Secreted Protease PrtA Controls Cell Growth, Biofilm Formation and Pathogenicity in Xylella fastidiosa
Source: Sci Rep. 2016 Aug 5;6:31098. doi: 10.1038/srep31098 (PMC4974619; doi:10.1038/srep31098)
Supplement: Supplementary Information [file srep31098-s1.pdf]

Supplementary Information for Gouran *et. al*. "A Secreted Protease PrtA Controls Cell Growth, Biofilm Formation and Pathogenicity in *Xylella fastidiosa*

Contents:

Figure S1. Genomic region of PD0956.

Figure S2. Disruption of PD0956 by insertion of a gentamicin resistance cassette and binary vector for the expression of PrtA in tobacco.

Figure S3. Western blot with anti-PrtA.

Table S1. Differentially expressed genes in the *prtA* mutant.

Table S2. Differentially expressed secreted proteins in the *prtA* mutant.

Table S3. Enrichment analysis of gene ontology terms from differentially expressed transcripts in the *prtA* mutant.

Complete Methods.

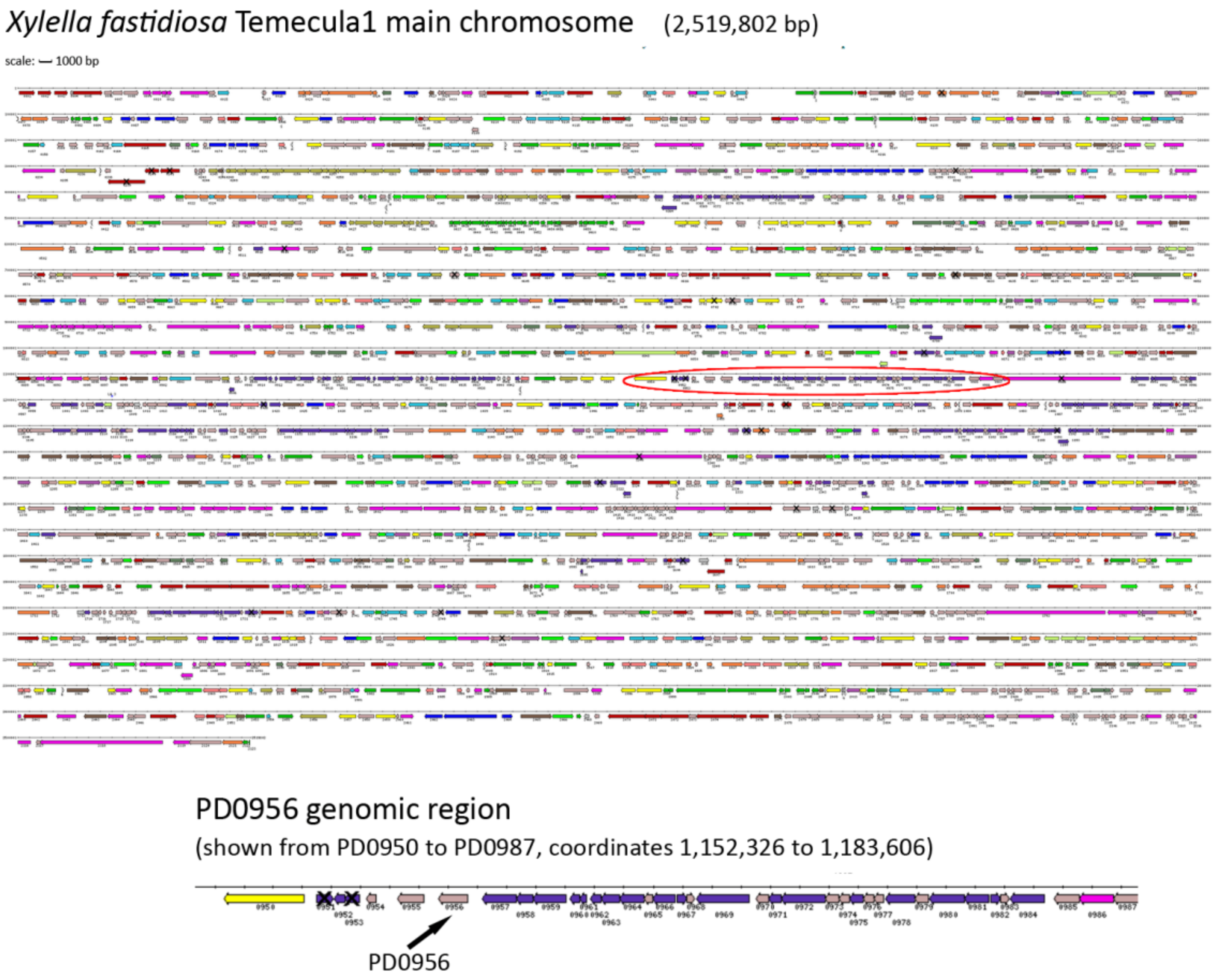

Figure S1. Genomic region of PD0956. Linear representation of the genome of *Xylella fastidiosa* Temecula1 strain, with protein, rRNA and tRNA coding sequences indicated by arrows according to their coding strand. Modified from http://bioinfo03.ibi.unicamp.br/lbi The region circled in red corresponding to genomic region spanning CDS PD0950 to PD0987 is shown in greater detail below, in which PD0956 is pointed. Downstream CDS PD0955 and PD0954 belong to separate transcriptional units according to the Prokaryotic Operon DB (http://operons.ibt.unam.mx/OperonPredictor/) and DOOR 2.0 server

(http://csbl.bmb.uga.edu/DOOR). Colors of arrows representing coding sequences are according to their functional COG groups as predicted during original genome annotation of strain Temecula1. In the detail yellow indicate macromolecule metabolism, blue indicates mobile genetic elements-related sequences, light brown indicate conserved hypothetical proteins and magenta indicate pathogenicity, virulence, and adaptation.

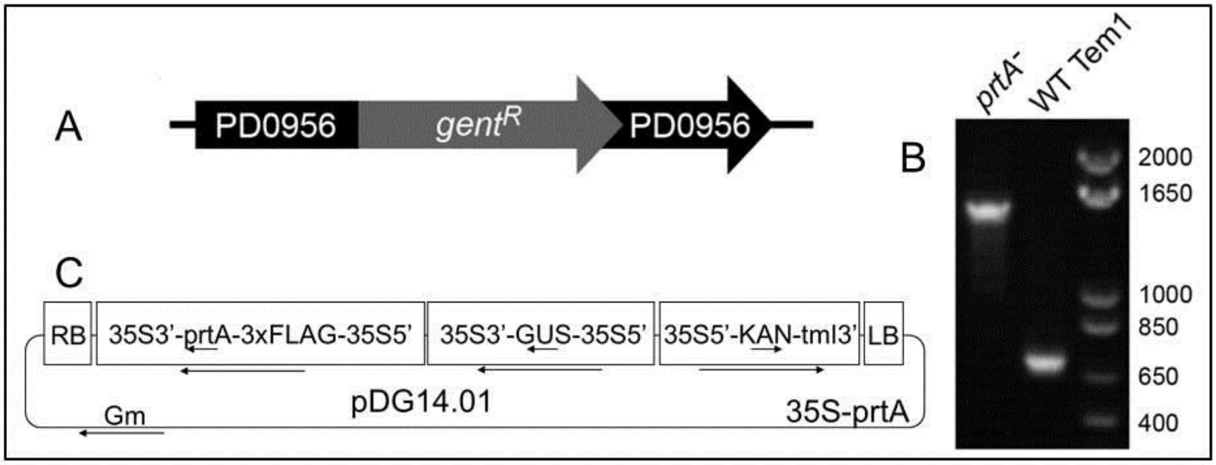

Figure S2. Disruption of PD0956 by insertion of a gentamicin resistance cassette.

(**A**) To disrupt PrtA (PD0956), a mutagenesis cassette was synthesized (GenScript, NJ) by inserting a gentamycin resistance gene within PrtA's aspartic acid residue at the coding region amino acid position 194, a predicted residue for the catalytic triad. The flanking homology region of this cassette was comprised of the entire open reading frame of PD0956 (581bp at 5' and 424bp at 3'). The synthesized pUC57-PD0956::Gm was electroporated into *X. fastidiosa* wild type Temecula1 as described previously[1]. (**B**) Transformant double crossover events were confirmed via gel electrophoresis and

sequencing using two primers, Fwd: GGCAGCCATGTGTTATCTGA and Rev: TCCCCCAGGAACAGTACAAC, designed to anneal to 5' and 3' regions of the PD0956. (**C**) **Binary vector for the expression of PrtA in tobacco.** Using epicenter Infusion Recombination cloning (Madison, WI), the PrtA sequence was cloned into our binary vector pDU92.3103 such that the coding region was downstream of the cauliflower mosaic virus (CaMV) promoter and upstream of the CaMV terminator. This resulted in binary vector pDG14.01.

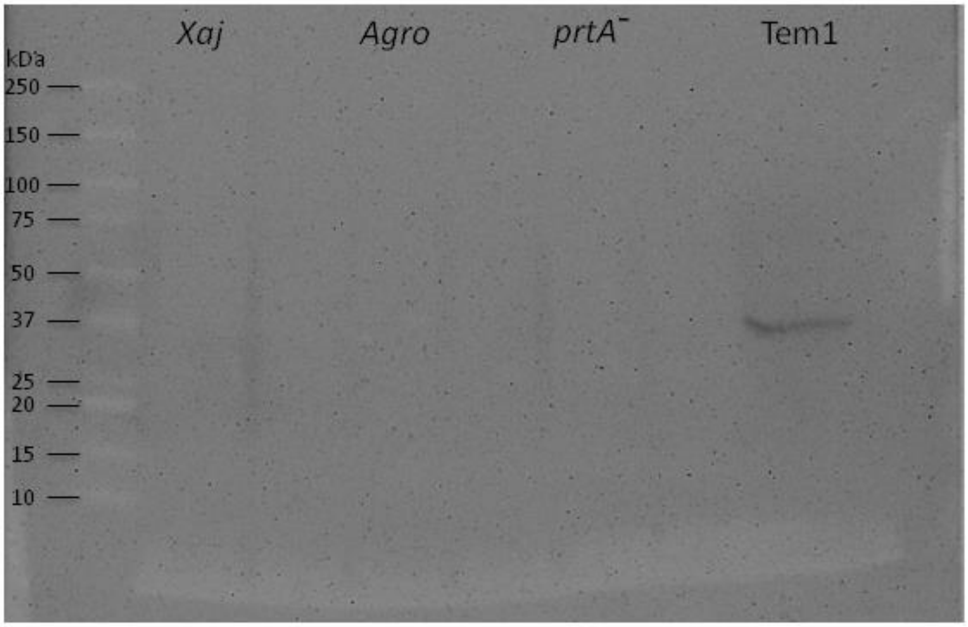

Figure S3. Western blot with anti-PrtA. Whole blot from which excerpt shown in Figure 2a was taken is shown, also containing protein extract from *Xanthomonas arboricola* pv. *juglandis* (*Xaj*) and *Agrobacterium tumefaciens* (*Agro*) besides the X. fastidiosa *prtA* mutant and wilt type Temecula1. See complete methods ahead.

Table S1. Differentially expressed genes in the *prtA* mutant.

Gene ID	GeneName	Description	log10 FC (*prtA*/WT)	P-Value
PD1526	-	tRNA-Ser	5.99	0.000
PD0050	-	tRNA-Ile	5.835	0.000
PD0135	-	tRNA-Ile	5.813	0.000
PD0096	-	tRNA-Ser	5.41	0.000
PD1609	-	tRNA-Asn	5.372	0.000
PD0411	-	tRNA-Leu	5.088	0.000
PD0340	-	tRNA-Glu	4.913	0.000
PD0191	-	tRNA-Met	4.88	0.000
PD0341	-	tRNA-Ala	4.815	0.000
PD0907	-	Uncharacterized protein	4.679	0.000
PD0184	-	tRNA-Glu	4.669	0.000
PD0477	-	tRNA-Asp	4.378	0.000
PD1880	-	tRNA-Gly	4.307	0.000
PD1085	-	Uncharacterized protein	4.154	0.000
Gent	-	Gentamicin resistance gene	4.142	0.000
PD1496	-	tRNA-Val	4.073	0.000
PD2017	-	tRNA-Gln	4.056	0.000
PD1060	-	Uncharacterized protein	4.026	0.000
PD0247	-	tRNA-Leu	3.868	0.000
PD1821	-	tRNA-Pro	3.844	0.000
PD2101	-	tRNA-His	3.767	0.000
PD0925	-	Phage-related protein	3.75	0.000
PD0237	-	tRNA-Phe	3.734	0.000
PD1733	-	tRNA-Val	3.66	0.000
PD0049	-	tRNA-Ala	3.593	0.000
PD1675	-	tRNA-Met	3.483	0.000
PD1176	-	Phage-related protein	3.44	0.000
PD1350	*trbN*	Conjugal transfer protein	3.39	0.000
PD1961	-	tRNA-Arg	3.364	0.000
PD0134	-	tRNA-Ala	3.347	0.000
Kan_R	-	Kanamycin resistance gene	3.275	0.000
PD2102	-	tRNA-Lys	3.104	0.000
PD0176	-	tRNA-Ala	2.901	0.000
PD1189	-	Uncharacterized protein	2.892	0.000
PD0789	-	Resolvase/integrase-like protein	2.888	0.000
PD0942	-	Uncharacterized protein	2.826	0.000
PD1349	-	Phage-related protein	2.818	0.000
PD2010	-	tRNA-Thr	2.796	0.000
PD0790	*traC*	DNA primase	2.785	0.000
PD1119	-	Phage-related protein	2.765	0.000
PD2099	-	tRNA-Pro	2.723	0.000
PD0510	-	tRNA-Lys	2.709	0.000

PD1378	*smpB*	SsrA-binding protein (Small protein B)	2.66	0.000
PD1191	-	Uncharacterized protein	2.655	0.000
PD0872	*isf*	Iron-sulfur flavoprotein	2.591	0.000
PD0047	-	tRNA-Arg	2.518	0.000
PD1409	*grx*	Glutaredoxin-like protein	2.483	0.000
PD0956	-	Uncharacterized protein	2.459	0.000
PD0944	-	tRNA-Lys	2.389	0.001
PD1215	-	Uncharacterized protein	2.382	0.000
PD0875	*paaK*	Frame shift	2.376	0.000
PD1343	*hicA*	HicA-related protein	2.337	0.000
PD1079	-	Alkylated DNA repair	2.317	0.000
PD0910	-	Uncharacterized protein	2.254	0.000
PD1327	-	Uncharacterized protein	2.237	0.000
PD0923	-	Phage-related protein	2.158	0.000
PD0974	-	Uncharacterized protein	2.146	0.000
PD1386	-	Response regulator	2.132	0.000
PD0913	-	Uncharacterized protein	2.114	0.000
PD1768	-	Threonylcarbamoyl-AMP synthase (TsaC)	2.104	0.000
PD2036	*purE*	N5-carboxyaminoimidazole ribonucleotide mutase (N5-CAIR mutase)	2.069	0.001
PD0936	-	Phage-related protein	2.065	0.000
PD0582	*aroK*	Shikimate kinase (SK) (EC 2.7.1.71)	2.053	0.000
PD0909	-	Uncharacterized protein	2.041	0.003
PD0929	-	Uncharacterized protein	2.038	0.000
PD0916	-	Uncharacterized protein	2.02	0.000
PD0926	-	Phage-related protein	2.012	0.000
PD1252	-	Uncharacterized protein	1.981	0.000
PD0125	-	Cysteine protease	1.953	0.000
PD2100	-	tRNA-Arg	1.952	0.001
PD0960	*parE*	Plasmid stabilization protein	1.944	0.000
PD0640	-	Lipopolysaccharide export system protein LptC	1.934	0.000
PD0912	-	Phage-related protein	1.912	0.003
PD0092	*lexA*	LexA repressor (EC 3.4.21.88)	1.9	0.000
PD1086	-	Uncharacterized protein	1.897	0.000
PD2105	-	Uncharacterized protein	1.845	0.000
PD0961	*parD*	Plasmid stabilization protein	1.839	0.000
PD1024	*amtB*	Ammonium transporter	1.798	0.000
PD1486	-	Uncharacterized protein	1.79	0.000
PD2011	-	tRNA-Gly	1.779	0.000
PD0922	-	Uncharacterized protein	1.768	0.004
PD0959	-	Phage-related protein	1.749	0.000
PD0932	-	Uncharacterized protein	1.741	0.000
PD0998	-	Uncharacterized protein	1.733	0.000
PD0388	*cls*	Cardiolipin synthase	1.732	0.000
PD0296	*proB*	Glutamate 5-kinase (Gamma-glutamyl kinase) (GK)	1.727	0.003
PD0931	-	Uncharacterized protein	1.692	0.000

PD0609	-	Uncharacterized protein	1.69	0.000
PD1400	-	Uncharacterized protein	1.684	0.000
PD0939	-	Phage-related protein	1.677	0.001
PD1161	-	Uncharacterized protein	1.66	0.002
PD0876	*trpD*	Anthranilate synthase component II	1.628	0.000
PD0877	*trpE*	Pseudogene	1.597	0.000
PD0085	*map*	Methionine aminopeptidase (MAP) (MetAP) (Peptidase M)	1.578	0.000
PD1074	-	Uncharacterized protein	1.55	0.006
PD0071	*ftsJ*	Ribosomal RNA large subunit methyltransferase E	1.538	0.000
PD1433	-	Fascin-like domain motif-containing	1.538	0.014
PD0898	*gpmA*	2,3-bisphosphoglycerate-dependent phosphoglycerate mutase (BPG-dependent PGAM)	1.523	0.000
PD1279	*dnaJ*	DnaJ protein	1.523	0.000
PD1334	-	IS1327 transposase	1.514	0.000
PD0906	-	Phage-related protein	1.505	0.000
PD0969	-	Phage-related protein	1.498	0.000
PD0935	-	Phage-related protein	1.494	0.000
PD1239	-	Uncharacterized protein	1.483	0.001
PD1819	-	Lipoprotein	1.445	0.000
PD1334	-	Uncharacterized protein	1.445	0.012
PD0587	-	Uncharacterized protein	1.434	0.001
PD0307	-	Toxin	1.426	0.004
PD0938	-	Phage-related protein	1.421	0.005
PD1831	-	Uncharacterized protein	1.416	0.015
PD1955	*pgpB*	Phosphatidylglycerophosphatase B	1.411	0.000
PD2109	-	Uncharacterized protein	1.404	0.016
PD1183	-	Anti-toxin protein	1.397	0.000
PD1146	*spsQ*	Glycosyl transferase	1.393	0.000
PD0955	-	Uncharacterized protein	1.365	0.001
PD0992	-	Phage-related protein	1.357	0.009
PD1908	-	tRNA-Pro	1.35	0.011
PD1177	-	Uncharacterized protein	1.328	0.000
PD1153	*algR*	Two-component system, regulatory protein	1.316	0.000
PD0235	-	Uncharacterized protein	1.313	0.000
PD1341	*vapI*	Virulence-associated protein	1.304	0.001
PD1159	-	Transcriptional regulator	1.296	0.001
PD1975	*ogt*	Methylated-DNA--protein-cysteine methyltransferase	1.295	0.000
PD0940	-	Uncharacterized protein	1.285	0.045
PD1861	*ftsZ*	Cell division protein FtsZ	1.274	0.000
PD0024	*pilE*	PilE protein	1.262	0.000
PD1830	-	Frame shift	1.248	0.000
PD0339	-	tRNA-Leu	1.244	0.014
PD0733	*xpsF*	General secretory pathway protein F	1.222	0.000
PD0599	*ctaA*	Cytochrome oxidase assembly protein	1.207	0.001
PD0470	-	tRNA-Leu	1.202	0.004

PD0370	-	Uncharacterized protein	1.193	0.000
PD0975	-	Phage-related protein	1.192	0.000
PD0982	-	Phage-related protein	1.186	0.000
PD1006	*fic*	Cell filamentation protein	1.181	0.001
PD1717	-	Uncharacterized protein	1.181	0.002
PD1825	-	Peptide methionine sulfoxide reductase MsrB	1.169	0.007
PD1815	-	Uncharacterized protein	1.165	0.001
PD2034	-	Glutaredoxin	1.161	0.001
PD0090	-	Uncharacterized protein	1.159	0.002
PD2021	*hemA*	Glutamyl-tRNA reductase (GluTR) (EC 1.2.1.70)	1.159	0.002
PD1689	*yjdB*	Inner membrane protein	1.156	0.000
PD1534	*thiD*	Phosphomethylpyrimidine kinase	1.156	0.001
PD1243	-	Uncharacterized protein	1.148	0.030
PD2048	*rpoH*	RNA polymerase sigma factor RpoH (RNA polymerase sigma-32 factor)	1.141	0.001
PD1023	*traE*	DNA topoisomerase (EC 5.99.1.2)	1.134	0.000
PD1668	-	Uncharacterized protein	1.134	0.000
PD0953	*traE*	Frame shift	1.133	0.000
PD1715	-	Uncharacterized protein	1.128	0.000
PD1803	-	Uncharacterized protein	1.114	0.003
PD1397	*leuB*	3-isopropylmalate dehydrogenase (EC 1.1.1.85) (3-IPM-DH)	1.113	0.000
PD0952	*traD*	Conjugal transfer protein	1.111	0.000
PD0708	-	Virulence regulator	1.111	0.000
PD0592	*htrB*	Lipid A biosynthesis lauroyl acyltransferase	1.094	0.000
PD0732	*xpsE*	General secretory pathway protein E	1.084	0.012
PD0075	-	Uncharacterized protein	1.082	0.001
PD1956	*ispG*	4-hydroxy-3-methylbut-2-en-1-yl diphosphate synthase (flavodoxin)	1.082	0.003
PD1676	-	tRNA-Gln	1.075	0.010
PD0712	-	Transposase	1.074	0.000
PD0796	-	Uncharacterized protein	1.059	0.002
PD1763	*def*	Peptide deformylase (PDF) (EC 3.5.1.88) (Polypeptide deformylase)	1.056	0.002
PD0673	-	ATPase	1.054	0.001
PD1686	-	Rubredoxin	1.053	0.049
PD2116	*pspA*	Hemagglutinin-like secreted protein	1.039	0.001
PD1005	-	Phage-related repressor protein	1.032	0.001
PD2077	*rhtC*	Amino acid transporter	1.029	0.001
PD1781	-	Endoribonuclease YbeY (EC 3.1.-.-)	1.027	0.000
PD1948	-	Uncharacterized protein	1.021	0.001
PD0829	-	Uncharacterized protein	1.02	0.001
PD1312	-	Uncharacterized protein	1.017	0.001
PD1853	-	Zinc protease	1.008	0.009
PD0871	-	Trp operon repressor homolog	1.002	0.009
PD0991	-	Phage-related protein	0.999	0.023
PD0148	*cvH*	Glycine cleavage system H protein	0.998	0.000
PD0329	*cdsA*	Phosphatidate cytidylyltransferase (EC	0.998	0.001

		2.7.7.41)		
PD1616	-	Uncharacterized protein	0.997	0.000
PD1533	-	Uncharacterized protein	0.996	0.024
PD1465	*malF*	ABC transporter sugar permease	0.989	0.005
PD1546	*mtfA*	Mannosyltransferase	0.988	0.001
PD0453	*rplR*	50S ribosomal protein L18	0.97	0.026
PD1184	-	Toxin-like protein	0.969	0.001
PD1439	*mviN*	Putative lipid II flippase MurJ	0.966	0.008
PD1467	-	Uncharacterized protein	0.963	0.015
PD1890	-	Beta-lactamase hydrolase-like protein (EC 3.-.-.-)	0.956	0.001
PD0054	-	Uncharacterized protein	0.95	0.001
PD0204	-	Uncharacterized protein	0.946	0.001
PD1642	*folB*	7,8-dihydroneopterin aldolase (EC 4.1.2.25)	0.945	0.003
PD1120	-	Phage-related protein	0.944	0.012
PD1067	-	Uncharacterized protein	0.94	0.005
PD0270	*oprO*	Polyphosphate-selective porin O	0.932	0.002
PD2106	-	Uncharacterized protein	0.931	0.014
PD0873	-	Uncharacterized protein	0.926	0.017
PD1431	-	Uncharacterized protein	0.917	0.022
PD0830	*yibK*	tRNA (cytidine/uridine-2'-O-)-methyltransferase TrmL	0.909	0.001
PD1840	*cysG*	Siroheme synthase	0.903	0.006
PD1408	-	Autolytic lysozyme	0.899	0.025
PD0314	-	Uncharacterized protein	0.898	0.006
PD1536	*cutA*	Periplasmic divalent cation tolerance protein	0.897	0.042
PD0917	-	Uncharacterized protein	0.893	0.004
PD0295	*argH*	Argininosuccinate lyase (ASAL) (EC 4.3.2.1) (Arginosuccinase)	0.893	0.013
PD0930	-	Phage-related protein	0.887	0.002
PD1418	-	Uncharacterized protein	0.883	0.026
PD1805	*yecS*	ABC transporter permease protein	0.882	0.002
PD0039	-	Uncharacterized protein	0.88	0.006
PD2047	*priA*	Primosomal protein N' (EC 3.6.4.-) (ATP-dependent helicase PriA)	0.875	0.002
PD1358	*serC*	Phosphoserine aminotransferase	0.874	0.003
PD0068	*folP*	Dihydropteroate synthase (DHPS) (EC 2.5.1.15) (Dihydropteroate pyrophosphorylase)	0.872	0.015
PD1800	*yadF*	Carbonic anhydrase (EC 4.2.1.1) (Carbonate dehydratase)	0.871	0.000
PD0098	*cysK*	Cysteine synthase	0.871	0.001
PD1924	*pilA*	Fimbrial protein	0.871	0.037
PD1371	*grpE*	Protein GrpE (HSP-70 cofactor)	0.866	0.018
PD0933	-	Phage-related protein	0.853	0.001
PD0361	*msbA*	Lipid A export ATP-binding/permease protein MsbA (EC 3.6.3.-)	0.853	0.005
PD0738	*pefK*	General secretory pathway protein K	0.851	0.005
PD0422	*wrbA*	Tryptophan repressor binding protein	0.851	0.010
PD0825	*ampE*	Transmembrane protein	0.849	0.002

PD1452	-	Lipopolysaccharide core biosynthesis protein	0.846	0.001
PD0426	*pheA*	Chorismate mutase (EC 5.4.99.5)	0.845	0.045
PD1787	-	Uncharacterized protein	0.838	0.004
PD1464	*malG*	ABC transporter sugar permease	0.836	0.002
PD1307	-	Uncharacterized protein	0.834	0.000
PD0305	*frpC*	Hemolysin-type calcium binding protein	0.83	0.024
PD0943	-	Phage-related protein	0.826	0.003
PD0126	-	Uncharacterized protein	0.822	0.009
PD1196	*int*	Phage-related integrase	0.816	0.001
PD1785	*corA*	Magnesium and cobalt transport protein	0.816	0.041
PD0529	*guxA*	Glucanase (EC 3.2.1.-)	0.81	0.009
PD0297	*proA*	Gamma-glutamyl phosphate reductase (GPR)	0.808	0.005
PD0323	*lpxA*	Acyl-[acyl-carrier-protein]--UDP-N-acetylglucosamine O-acyltransferase	0.807	0.023
PD0562	*queA*	S-adenosylmethionine:tRNA ribosyltransferase-isomerase	0.806	0.032
PD1125	-	Uncharacterized protein	0.805	0.019
PD1076	-	Uncharacterized protein	0.803	0.012
PD0362	*lpxK*	Tetraacyldisaccharide 4'-kinase (EC 2.7.1.130) (Lipid A 4'-kinase)	0.802	0.002
PD1349	-	Conjugal transfer protein	0.801	0.017
PD1611	*pilY1*	Type IV pilus biogenesis factor PilY1 homolog PD_1611	0.8	0.008
PD0610	*truA*	tRNA pseudouridine synthase A (EC 5.4.99.12)	0.798	0.008
PD0045	-	Uncharacterized protein	0.798	0.013
PD1735	*fimT*	Type 4 fimbrial biogenesis protein	0.796	0.014
PD1525	-	Peptidyl-prolyl cis-trans isomerase (EC 5.2.1.8)	0.795	0.005
PD1438	*ribF*	Riboflavin biosynthesis protein (EC 2.7.1.26) (EC 2.7.7.2)	0.793	0.010
PD2113	-	Uncharacterized protein	0.787	0.032
PD0032	-	Uncharacterized protein	0.784	0.016
PD2041	*rep*	ATP-dependent DNA helicase Rep (EC 3.6.4.12)	0.781	0.003
PD1169	*btuE*	Glutathione peroxidase	0.779	0.044
PD1253	*efp*	Elongation factor P-like protein	0.778	0.024
PD0818	*yneN*	Thioredoxin	0.777	0.015
PD1681	-	tRNA-dihydrouridine(20/20a) synthase	0.775	0.011
PD1604	-	Uncharacterized protein	0.774	0.017
PD1228	-	Uncharacterized protein	0.772	0.008
PD0879	-	Uncharacterized protein	0.771	0.002
PD1151	-	Uncharacterized protein	0.77	0.005
PD1817	*surE*	5'-nucleotidase SurE (EC 3.1.3.5) (Nucleoside 5'-monophosphate phosphohydrolase)	0.768	0.032
PD1100	-	Uncharacterized protein	0.766	0.006
PD2104	-	Uncharacterized protein	0.766	0.010
PD1690	-	Transmembrane protein	0.761	0.006
PD0070	*hflB*	ATP-dependent zinc metalloprotease FtsH (EC 3.4.24.-)	0.76	0.035
PD0561	*mrdB*	Rod shape-determining protein	0.759	0.003

PD2110	*pspA*	Hemagglutinin-like secreted protein	0.758	0.006
PD0093	*recA*	Protein RecA (Recombinase A)	0.755	0.001
PD1769	*lpxA*	Acyl-[ACP]-UDP-N-acetylglucosamine	0.754	0.006
PD0899	*nfi*	Endonuclease V (DNase V)	0.751	0.006
PD1957	*actS*	Two-component system, sensor protein	0.751	0.011
PD1466	*malE*	ABC transporter sugar-binding protein	0.75	0.001
PD0928	-	Phage-related protein	0.744	0.002
PD0139	-	Uncharacterized protein	0.744	0.004
PD0669	*trxB*	Thioredoxin reductase (EC 1.8.1.9)	0.74	0.006
PD0945	*exsB*	7-cyano-7-deazaguanine synthase (EC 6.3.4.20)	0.74	0.014
PD1139	*int*	Phage-related integrase	0.737	0.000
PD1374	*fur*	Ferric uptake regulator	0.736	0.008
PD0332	*fadL*	Fatty acid outer membrane porin	0.736	0.027
PD0677	*ptr1*	Pteridine reductase 1	0.735	0.035
PD0535	-	Uncharacterized protein	0.732	0.020
PD0646	-	Uncharacterized protein	0.731	0.002
PD0378	*gp4*	Phage-related portal protein	0.727	0.010
PD0703	-	Pseudogene	0.725	0.011
PD0574	*cybB*	Cytochrome B561	0.722	0.002
PD1820	*nlpD*	Lipoprotein	0.718	0.004
PD1135	-	Uncharacterized protein	0.715	0.024
PD0386	-	Uncharacterized protein	0.714	0.004
PD0380	-	Phage-related protein	0.711	0.018
PD1804	*metN*	Methionine import ATP-binding protein MetN (EC 3.6.3.-)	0.71	0.005
PD1797	-	Uncharacterized protein	0.706	0.046
PD0954	-	Uncharacterized protein	0.705	0.024
PD0915	-	Uncharacterized protein	0.704	0.005
PD0444	*rplP*	50S ribosomal protein L16	0.704	0.015
PD0679	-	Uncharacterized protein	0.704	0.023
PD0434	-	ATP synthase subunit a (ATP synthase F0 sector subunit a) (F-ATPase subunit 6)	0.699	0.010
PD0608	*asd*	Aspartate-semialdehyde dehydrogenase (ASA dehydrogenase)	0.697	0.028
PD1627	*purA*	Adenylosuccinate synthetase (AMPSase) (IMP--aspartate ligase)	0.696	0.006
PD0578	-	Uncharacterized protein	0.694	0.007
PD2072	*hsdM*	Type I restriction-modification system DNA methylase	0.694	0.014
PD0967	-	Phage-related protein	0.692	0.010
PD0076	-	Glycosyl transferase	0.691	0.006
PD1062	-	Uncharacterized protein	0.691	0.010
PD1322	*dpoL*	Phage-related DNA polymerase	0.691	0.011
PD1192	-	Uncharacterized protein	0.689	0.036
PD1010	-	Uncharacterized protein	0.687	0.003
PD0520	*attO*	Transcriptional regulator AraC family	0.687	0.030
PD0667	*aat*	Leucyl/phenylalanyl-tRNA--protein transferase	0.684	0.009
PD0850	-	Uncharacterized protein	0.684	0.025

PD0145	*yjjV*	Deoxyribonuclease	0.679	0.008
PD0315	*gaa*	Glutaryl-7-ACA acylase	0.677	0.008
PD0869	*nadA*	Quinolinate synthase A (EC 2.5.1.72)	0.677	0.019
PD0309	*ate1*	Putative arginyl-tRNA--protein transferase (Arginyltransferase)	0.672	0.030
PD1080	-	Uncharacterized protein	0.67	0.005
PD1558	*comE*	DNA transport competence protein	0.668	0.007
PD0122	*pyrE*	Orotate phosphoribosyltransferase (OPRT) (OPRTase) (EC 2.4.2.10)	0.668	0.014
PD0678	-	Uncharacterized protein	0.666	0.020
PD0777	*aspH*	Aspartyl/asparaginyl beta-hydroxylase	0.657	0.005
PD1235	*mltB*	Membrane-bound lytic transglycosylase	0.652	0.022
PD0097	*prlC*	Oligopeptidase A	0.651	0.036
PD0384	*int*	Phage-related integrase	0.65	0.003
PD1163	-	Fructokinase	0.65	0.015
PD1296	*acs*	Acetyl-coenzyme A synthetase (AcCoA synthetase)	0.649	0.024
PD0990	*int*	Phage-related integrase	0.648	0.047
PD1257	*potE*	Cationic amino acid transporter	0.647	0.008
PD1204	*pstA*	Phosphate transport system permease protein PstA	0.646	0.019
PD1459	-	Uncharacterized protein	0.642	0.007
PD1042	*prc*	Tail-specific protease	0.642	0.014
PD0117	*radC*	UPF0758 protein PD_0117	0.641	0.018
PD0803	*pepP*	Aminopeptidase P	0.638	0.015
PD1855	-	Uncharacterized protein	0.633	0.006
PD0843	*tonB*	TonB protein	0.632	0.007
PD0653	-	Ion transporter 33.9 kDa	0.629	0.007
PD1641	-	Uncharacterized protein	0.628	0.007
PD0720	*gmk*	Guanylate kinase (EC 2.7.4.8) (GMP kinase)	0.628	0.042
PD0997	-	Uncharacterized protein	0.626	0.006
PD1749	-	Frame shift	0.625	0.039
PD1308	*metE*	5-methyltetrahydropteroyltriglutamate--homocysteine methyltransferase	0.615	0.017
PD1209	*apaG*	Protein ApaG	0.614	0.033
PD0709	-	UPF0162 protein PD_0709	0.608	0.026
PD0418	*tldD*	TldD protein	0.607	0.033
PD1728	-	Phage-related protein	0.607	0.046
PD0648	-	Uncharacterized protein	0.606	0.015
PD1224	-	Uncharacterized protein	0.606	0.030
PD0604	*psd*	Phosphatidylserine decarboxylase proenzyme (EC 4.1.1.65)	0.606	0.038
PD0150	-	Uncharacterized protein	0.605	0.025
PD0645	-	Uncharacterized protein	0.604	0.013
PD0146	-	Outer membrane protein	0.601	0.031
PD1954	-	Uncharacterized protein	0.6	0.012
PD1043	*ilvC*	Ketol-acid reductoisomerase	0.599	0.038
PD1892	-	UPF0394 membrane protein PD_1892	0.597	0.043
PD0501	*yadG*	ABC transporter ATP-binding protein	0.595	0.039

PD1783	*corC*	Magnesium and cobalt efflux protein	0.593	0.050
PD0069	-	Uncharacterized protein	0.588	0.037
PD2079	-	Uncharacterized protein	0.586	0.009
PD0711	*yncD*	TonB-dependent receptor	0.586	0.012
PD1342	*hicB*	HicB-related protein	0.582	0.022
PD0671	-	Uncharacterized protein	0.581	0.010
PD0701	*hslV*	ATP-dependent protease subunit HslV (EC 3.4.25.2)	0.579	0.031
PD2075	*hsdS*	Type I restriction-modification system specificity determinant	0.57	0.017
PD0576	-	Histidine kinase/response regulator hybrid protein	0.569	0.010
PD1692	*pilP*	Fimbrial assembly protein	0.566	0.021
PD0445	*rpmC*	50S ribosomal protein L29	0.565	0.032
PD1794	-	Uncharacterized protein	0.563	0.045
PD1818	*pcm*	Protein-L-isoaspartate O-methyltransferase	0.559	0.007
PD1149	-	Transcriptional regulator	0.559	0.018
PD1929	*pilS*	Two-component system, sensor protein	0.543	0.029
PD1643	*gcp*	tRNA N6-adenosine threonylcarbamoyltransferase	0.541	0.021
PD1949	*rfbD*	Transport permease protein	0.541	0.035
PD1771	-	Uncharacterized protein	0.54	0.048
PD0676	*folK*	2-amino-4-hydroxy-6-hydroxymethyldihydropteridine pyrophosphokinase	0.536	0.020
PD0591	*cysA*	Sulfate/thiosulfate import ATP-binding protein CysA	0.536	0.040
PD0532	-	UPF0250 protein PD_0532	0.529	0.036
PD0767	-	Uncharacterized protein	0.527	0.017
PD0797	*rpiA*	Ribose-5-phosphate isomerase A (EC 5.3.1.6) (Phosphoriboisomerase A) (PRI)	0.526	0.023
PD0261	*nuoN*	NADH-quinone oxidoreductase subunit N	0.522	0.031
PD1129	-	Uncharacterized protein	0.52	0.045
PD0564	-	Uncharacterized protein	0.518	0.047
PD0163	-	Uncharacterized protein	0.514	0.039
PD1158	-	Uncharacterized protein	0.514	0.046
PD2059	-	Peptidase	0.512	0.042
PD1683	-	Uncharacterized protein	0.509	0.022
PD0889	*ruvB*	Holliday junction ATP-dependent DNA helicase RuvB (EC 3.6.4.12)	0.509	0.037
PD0387	-	Uncharacterized protein	0.509	0.045
PD0023	*pilY1*	Type IV pilus biogenesis factor PilY1 homolog PD_0023	0.503	0.038
PD1401	*queF*	NADPH-dependent 7-cyano-7-deazaguanine reductase	0.503	0.043
PD2022	*prfA*	Peptide chain release factor 1 (RF-1)	0.494	0.023
PD0721	-	Alpha helix protein	0.491	0.046
PD0865	-	Frame shift	0.49	0.026
PD1917	-	Uncharacterized protein	0.489	0.019
PD1976	*enpP*	Phosphodiesterase-nucleotide pyrophosphatase	0.476	0.035

PD0702	*hslU*	Pseudogene	0.475	0.045
PD1154	*algZ*	Two-component system, sensor protein	0.466	0.050
PD1503	*fabG*	3-oxoacyl-[ACP] reductase	0.463	0.048
PD0328	*dxr*	1-deoxy-D-xylulose 5-phosphate reductoisomerase (DXP reductoisomerase)	0.453	0.047
PD1200	*bfeA*	Ferric enterobactin receptor	0.447	0.042
PD1152	*hemC*	Porphobilinogen deaminase (PBG)	0.44	0.039
PD1451	*capD*	Epimerase/dehydratase protein	0.433	0.043
PD1832	-	Uncharacterized antigen	0.417	0.050
PD0089	*asnB*	Asparagine synthase B	-0.437	0.043
PD1742	*thiL*	Thiamine-monophosphate kinase (TMP kinase)	-0.448	0.039
PD1548	*cysM*	Cystathionine beta-synthase	-0.452	0.048
PD0621	*cyoD*	Cytochrome O ubiquinol oxidase subunit IV	-0.457	0.047
PD0742	*xpsD*	General secretory pathway protein D	-0.461	0.038
PD0840	*glyQ*	Glycine--tRNA ligase alpha subunit (EC 6.1.1.14)	-0.463	0.039
PD1859	-	Uncharacterized protein	-0.471	0.036
PD0987	-	Uncharacterized protein	-0.481	0.037
PD0319	*ahcY*	Adenosylhomocysteinase (EC 3.3.1.1)	-0.489	0.037
PD1834	*pdxA*	4-hydroxythreonine-4-phosphate dehydrogenase	-0.499	0.037
PD1968	*ppk*	Polyphosphate kinase (EC 2.7.4.1)	-0.506	0.025
PD0281	*pyrC*	Dihydroorotase (EC 3.5.2.3)	-0.513	0.020
PD0333	-	Uncharacterized protein	-0.515	0.043
PD0061	-	Chaperone protein	-0.52	0.040
PD1270	*hisS*	Histidine--tRNA ligase (EC 6.1.1.21)	-0.525	0.031
PD1213	-	3-deoxy-D-manno-octulosonic acid kinase (Kdo kinase) (EC 2.7.1.166)	-0.526	0.016
PD0947	*fumB*	Fumarate hydratase	-0.526	0.026
PD1729	-	Phage-related protein	-0.527	0.021
PD1379	-	Uncharacterized protein	-0.529	0.027
PD1886	-	Uncharacterized protein	-0.529	0.050
PD1896	-	Uncharacterized protein	-0.537	0.038
PD0236	*acnB*	Aconitate hydratase B (EC 4.2.1.3)	-0.537	0.039
PD0086	*dapD*	2,3,4,5-tetrahydropyridine-2,6-dicarboxylate N-succinyltransferase	-0.541	0.028
PD0414	*omp28*	Outer membrane protein	-0.541	0.038
PD0615	-	Luciferase	-0.546	0.050
PD0442	*rplV*	50S ribosomal protein L22	-0.548	0.044
PD2016	*prsA*	Ribose-phosphate pyrophosphokinase (RPPK)	-0.549	0.046
PD0441	*rpsS*	30S ribosomal protein S19	-0.549	0.046
PD2062	*gltD*	Glutamate synthase, beta subunit	-0.552	0.028
PD0120	*algC*	Phosphomannomutase	-0.553	0.009
PD1286	*mucD*	Periplasmic protease	-0.553	0.023
PD0420	*pmbA*	PmbA protein	-0.553	0.028
PD2057	*ctpA*	Carboxyl-terminal protease	-0.555	0.036
PD0353	*sdhB*	Succinate dehydrogenase iron-sulfur subunit (EC 1.3.5.1)	-0.557	0.048
PD0381	-	Phage-related protein	-0.559	0.021

PD2037	-	Uncharacterized protein	-0.56	0.019
PD2007	*secE*	Preprotein translocase SecE subunit	-0.561	0.009
PD1701	*dnaB*	Replicative DNA helicase (EC 3.6.4.12)	-0.565	0.023
PD1862	*ftsA*	Cell division protein FtsA	-0.566	0.033
PD2071	*hsdS*	Type I restriction-modification system specificity determinant	-0.567	0.021
PD0543	*eno*	Enolase (EC 4.2.1.11)	-0.579	0.048
PD0228	-	Uncharacterized protein	-0.583	0.013
PD0174	-	Uncharacterized protein	-0.583	0.028
PD1225	-	Uncharacterized protein	-0.584	0.018
PD1635	*proS*	Proline--tRNA ligase (EC 6.1.1.15) (Prolyl-tRNA synthetase) (ProRS)	-0.587	0.014
PD1234	-	Ribosomal silencing factor RsfS	-0.588	0.018
PD0110	*glmS*	Glutamine--fructose-6-phosphate aminotransferase	-0.592	0.043
PD0685	*accD*	Acetyl-coenzyme A carboxylase carboxyl transferase subunit beta	-0.593	0.034
PD0823	*folE*	GTP cyclohydrolase 1 (EC 3.5.4.16)	-0.598	0.007
PD0490	*cls*	Cardiolipin synthase	-0.598	0.022
PD0399	*carB*	Carbamoyl-phosphate synthase large chain (EC 6.3.5.5)	-0.604	0.041
PD1659	*vacJ*	Lipoprotein	-0.605	0.015
PD1695	*pilM*	Fimbrial assembly membrane protein	-0.611	0.025
PD0606	*yfcB*	50S ribosomal protein L3 glutamine methyltransferase (L3 MTase)	-0.613	0.017
PD0001	*dnaA*	Chromosomal replication initiator protein DnaA	-0.615	0.037
PD0496	*cvaA*	Colicin V secretion protein	-0.617	0.026
PD0033	-	Transcription-repair-coupling factor (TRCF)	-0.618	0.027
PD1293	*dxs*	1-deoxy-D-xylulose-5-phosphate synthase (EC 2.2.1.7)	-0.619	0.025
PD0881	*ftsY*	Signal recognition particle receptor FtsY (SRP receptor)	-0.619	0.030
PD1935	*gyrA*	DNA gyrase subunit A (EC 5.99.1.3)	-0.62	0.022
PD0620	*gcvP*	Glycine dehydrogenase (decarboxylating)	-0.621	0.020
PD2023	*rlmL*	Ribosomal RNA large subunit methyltransferase K/L	-0.621	0.041
PD0282	-	Putative membrane protein insertion efficiency factor	-0.623	0.013
PD1751	*yjjK*	ABC transporter ATP-binding protein	-0.623	0.013
PD0344	*pgl*	6-phosphogluconolactonase (6PGL) (EC 3.1.1.31)	-0.623	0.018
PD1022	-	Uncharacterized protein	-0.625	0.010
PD1406	*yahK*	Alcohol dehydrogenase	-0.627	0.029
PD1654	*ttg2A*	Toluene tolerance protein	-0.632	0.014
PD0461	*rpoA*	DNA-directed RNA polymerase subunit alpha (RNAP subunit alpha)	-0.632	0.046
PD0112	*speE*	Polyamine aminopropyltransferase (Putrescine aminopropyltransferase)	-0.634	0.017
PD1972	*grxC*	Glutaredoxin	-0.634	0.017
PD0784	*acrA*	Precursor of drug resistance protein	-0.638	0.012
PD0038	*hmpA*	Flavohemoprotein (Flavohemoglobin)	-0.639	0.023

		(Hemoglobin-like protein) (Nitric oxide dioxygenase)		
PD2053	*rhlB*	ATP-dependent RNA helicase RhlB (EC 3.6.4.13)	-0.643	0.020
PD0798	-	Uncharacterized protein	-0.645	0.010
PD1569	*tmk*	Thymidylate kinase (EC 2.7.4.9) (dTMP kinase)	-0.645	0.034
PD1470	-	Segregation and condensation protein B	-0.646	0.006
PD0832	*fabH*	3-oxoacyl-[ACP] synthase III	-0.646	0.023
PD0198	*pnp*	Polyribonucleotide nucleotidyltransferase (EC 2.7.7.8) (Polynucleotide phosphorylase) (PNPase)	-0.647	0.040
PD1387	*gumM*	GumM protein	-0.648	0.029
PD1766	-	Protein Smg homolog	-0.649	0.036
PD1746	*ribC*	Riboflavin synthase alpha chain	-0.65	0.019
PD1823	*alr*	Alanine racemase (EC 5.1.1.1)	-0.652	0.011
PD0985	-	Uncharacterized protein	-0.653	0.011
PD1912	*pheS*	Phenylalanine--tRNA ligase alpha subunit (EC 6.1.1.20) (PheRS)	-0.661	0.006
PD0437	*rplC*	50S ribosomal protein L3	-0.661	0.028
PD1277	*dacC*	Penicillin-binding protein 6	-0.663	0.016
PD0792	-	Uncharacterized protein	-0.665	0.024
PD1910	*ihfA*	Integration host factor subunit alpha (IHF-alpha)	-0.667	0.003
PD1744	*ribH*	6,7-dimethyl-8-ribityllumazine synthase (DMRL synthase) (LS) (Lumazine synthase) (EC 2.5.1.78)	-0.668	0.006
PD1684	-	Uncharacterized protein	-0.668	0.015
PD1980	*phhB*	Putative pterin-4-alpha-carbinolamine dehydratase (PHS)	-0.67	0.007
PD0697	-	Uncharacterized protein	-0.67	0.016
PD1606	-	Uncharacterized protein	-0.673	0.027
PD2015	*rplY*	50S ribosomal protein L25 (General stress protein CTC)	-0.677	0.007
PD0250	*nuoC*	NADH-quinone oxidoreductase subunit C (EC 1.6.5.11) (NADH dehydrogenase I subunit C)	-0.677	0.013
PD0246	*secG*	Protein-export membrane protein SecG	-0.679	0.006
PD1849	*zur*	Transcriptional regulator Fur family	-0.681	0.012
PD0783	*acrF*	Multidrug-efflux transporter	-0.684	0.015
PD0245	*tpiA*	Triosephosphate isomerase (TIM) (EC 5.3.1.1) (Triose-phosphate isomerase)	-0.693	0.002
PD1554	-	Uncharacterized protein	-0.697	0.027
PD0058	*mrkD*	Fimbrial adhesin	-0.701	0.013
PD0651	*dsbC*	Disulfide isomerase	-0.702	0.023
PD1096	*I*	Phage-related tail protein	-0.703	0.039
PD0725	-	Uncharacterized protein	-0.704	0.009
PD0584	*glnS*	Glutamine--tRNA ligase (EC 6.1.1.18) (Glutaminyl-tRNA synthetase) (GlnRS)	-0.705	0.009
PD1587	-	Uncharacterized protein	-0.705	0.023
PD1662	*nrtD*	Nitrate ABC transporter ATP-binding protein	-0.706	0.006
PD0082	*rimM*	Ribosome maturation factor RimM	-0.71	0.022
PD0197	*rpsO*	30S ribosomal protein S15	-0.713	0.008

PD1573	-	tRNA 2-thiocytidine biosynthesis protein TtcA	-0.715	0.006
PD0081	*rpsP*	30S ribosomal protein S16	-0.717	0.009
PD1321	-	Phage-related protein	-0.717	0.042
PD0487	-	Uncharacterized protein	-0.718	0.007
PD1490	*efp*	Elongation factor P (EF-P)	-0.721	0.015
PD2006	*nusG*	Transcription termination/antitermination protein NusG	-0.721	0.020
PD0401	*rpfE*	Regulatory protein	-0.723	0.003
PD1329	-	Uncharacterized protein	-0.724	0.003
PD0553	-	ABC transporter ATP-binding protein	-0.724	0.004
PD1809	*pdhB*	Acetyltransferase component of pyruvate dehydrogenase complex (EC 2.3.1.12)	-0.725	0.003
PD0986	*pspA*	Hemagglutinin-like secreted protein	-0.729	0.013
PD1185	-	Phage-related protein	-0.73	0.003
PD1014	-	Phage-related protein	-0.738	0.037
PD0160	-	Uncharacterized protein	-0.741	0.004
PD1747	*ribD*	Riboflavin biosynthesis protein RibD	-0.741	0.038
PD1061	*dinD*	DNA-damage-inducible protein D	-0.743	0.002
PD1745	*ribA*	GTP cyclohydrolase II	-0.743	0.005
PD1669	-	Uncharacterized protein	-0.744	0.007
PD1672	*bfr*	Bacterioferritin	-0.744	0.031
PD0820	*vacB*	Ribonuclease R (RNase R) (EC 3.1.13.1)	-0.745	0.004
PD0518	-	UPF0192 protein PD_0518	-0.748	0.006
PD1628	-	Uncharacterized protein	-0.752	0.016
PD0759	*sucB*	Dihydrolipoyllysine-residue succinyltransferase component of 2-oxoglutarate dehydrogenase complex	-0.759	0.005
PD1998	*rpsG*	30S ribosomal protein S7	-0.76	0.004
PD0398	*carA*	Carbamoyl-phosphate synthase small chain (EC 6.3.5.5)	-0.76	0.009
PD0351	*sdhD*	Succinate dehydrogenase hydrophobic membrane anchor subunit	-0.762	0.001
PD0065	-	GTPase HflX (GTP-binding protein HflX)	-0.762	0.004
PD0310	-	Pathogenicity-related protein	-0.764	0.007
PD1116	-	Phage-related protein	-0.765	0.014
PD0557	*mreB*	Rod shape-determining protein	-0.769	0.001
PD0650	*purL*	Phosphoribosylformylglycinamidine synthase (FGAM synthase)	-0.77	0.004
PD1513	*xseB*	Exodeoxyribonuclease 7 small subunit (EC 3.1.11.6)	-0.77	0.036
PD1457	*cmk*	Cytidylate kinase (CK) (EC 2.7.4.25) (Cytidine monophosphate kinase) (CMP kinase)	-0.774	0.013
PD1657	*ttg2D*	Toluene tolerance protein	-0.775	0.001
PD0542	*kdsA*	2-dehydro-3-deoxyphosphooctonate aldolase (EC 2.5.1.55)	-0.775	0.003
PD1456	*rpsA*	30S ribosomal protein S1	-0.776	0.003
PD1621	-	Uncharacterized protein	-0.778	0.008
PD1287	*lepA*	Elongation factor 4 (EF-4) (EC 3.6.5.n1) (Ribosomal back-translocase LepA)	-0.779	0.010
PD0833	*mod*	Methyltransferase, type III restriction-modification system	-0.78	0.003

PD0768	-	Phage-related protein	-0.783	0.037
PD0088	*dapE*	Succinyl-diaminopimelate desuccinylase (SDAP desuccinylase)	-0.784	0.022
PD1658	-	Uncharacterized protein	-0.785	0.001
PD1443	*rplU*	50S ribosomal protein L21	-0.786	0.001
PD2033	-	UPF0056 inner membrane protein	-0.786	0.009
PD0530	*lipA*	Lipoyl synthase (EC 2.8.1.8) (Lip-syn) (LS) (Lipoate synthase)	-0.788	0.003
PD0436	*rpsJ*	30S ribosomal protein S10	-0.788	0.009
PD0698	*dapF*	Diaminopimelate epimerase (DAP epimerase) (EC 5.1.1.7)	-0.79	0.026
PD1297	-	Putative manganese efflux pump MntP	-0.79	0.026
PD0175	*prsX*	Transcriptional regulator MarR family	-0.792	0.022
PD0402	*recJ*	Single-stranded DNA exonuclease	-0.793	0.004
PD1572	*fabA*	3-hydroxydecanoyl-[acyl-carrier-protein] dehydratase (EC 4.2.1.59)	-0.797	0.004
PD0705	*pepN*	Aminopeptidase N	-0.797	0.005
PD0164	*accA*	Acetyl-coenzyme A carboxylase carboxyl transferase subunit alpha (ACCase subunit alpha)	-0.801	0.022
PD0686	*glmM*	Phosphoglucosamine mutase (EC 5.4.2.10)	-0.804	0.015
PD1865	*murC*	UDP-N-acetylmuramate--L-alanine ligase (EC 6.3.2.8)	-0.804	0.021
PD1586	-	UPF0102 protein PD_1586	-0.805	0.012
PD1226	*ppa*	Inorganic pyrophosphatase (EC 3.6.1.1) (Pyrophosphate phospho-hydrolase) (PPase)	-0.811	0.004
PD0746	*ahpF*	Subunit F of alkyl hydroperoxide reductase	-0.819	0.005
PD0300	-	Uncharacterized protein	-0.824	0.012
PD0465	*lip*	Triacylglycerol lipase	-0.833	0.007
PD0760	*sucA*	Oxoglutarate dehydrogenase	-0.84	0.003
PD0313	*pspB*	Serine protease	-0.841	0.006
PD0494	*typA*	GTP-binding elongation factor protein	-0.852	0.006
PD0142	*prfC*	Peptide chain release factor 3 (RF-3)	-0.855	0.002
PD1317	-	Uncharacterized protein	-0.857	0.032
PD0763	*fumC*	Fumarate hydratase class II (Fumarase C) (EC 4.2.1.2)	-0.859	0.001
PD0629	*gloA*	Lactoylglutathione lyase (EC 4.4.1.5) (Glyoxalase I)	-0.863	0.006
PD0218	*pspB*	Serine protease	-0.865	0.002
PD0207	*etfB*	Electron transfer flavoprotein beta subunit	-0.865	0.010
PD1440	*rpsT*	30S ribosomal protein S20	-0.868	0.006
PD1807	*ompW*	Outer membrane protein	-0.877	0.010
PD0757	*pcp*	Peptidoglycan-associated outer membrane lipoprotein	-0.878	0.007
PD1579	*tatB*	Sec-independent protein translocase protein TatB	-0.879	0.007
PD0458	*rpsM*	30S ribosomal protein S13	-0.88	0.014
PD0448	*rplX*	50S ribosomal protein L24	-0.881	0.007
PD1088	*D*	Phage-related tail protein	-0.883	0.005
PD1636	-	Uncharacterized protein	-0.887	0.001
PD1549	*uptF*	UptF protein	-0.893	0.003

PD1582	*guaA*	Glutamine amidotransferase	-0.896	0.018
PD0563	*relA*	ATP:GTP 3'-pyrophosphotranferase	-0.897	0.001
PD1922	*pilD*	Type 4 prepilin-like proteins leader peptide-processing enzyme (EC 3.4.23.43)	-0.901	0.000
PD1971	-	Putative beta-barrel assembly-enhancing protease (EC 3.4.-.-)	-0.907	0.002
PD1031	-	Uncharacterized protein	-0.91	0.016
PD1791	-	Uncharacterized protein	-0.911	0.002
PD0613	*trpA*	Tryptophan synthase alpha chain (EC 4.2.1.20)	-0.913	0.000
PD1914	*rpmI*	50S ribosomal protein L35	-0.913	0.024
PD1157	*pbp*	Beta-lactamase-like protein	-0.915	0.000
PD1301	-	Oligopeptide transporter	-0.921	0.000
PD1720	-	Uncharacterized protein	-0.923	0.002
PD0376	-	Uncharacterized protein	-0.925	0.009
PD0365	*I*	Phage-related tail protein	-0.926	0.002
PD0612	*trpB*	Tryptophan synthase beta chain (EC 4.2.1.20)	-0.927	0.000
PD0221	*pfkA*	Pyrophosphate--fructose 6-phosphate 1-phosphotransferase (EC 2.7.1.90)	-0.927	0.002
PD0447	*rplN*	50S ribosomal protein L14	-0.928	0.002
PD0289	-	Uncharacterized protein	-0.932	0.023
PD0277	-	Ferredoxin	-0.936	0.012
PD1346	-	Uncharacterized protein	-0.937	0.001
PD0215	*cvaC*	Colicin V	-0.941	0.019
PD0379	*dpoL*	Phage-related DNA polymerase	-0.941	0.027
PD2092	-	Uncharacterized protein	-0.949	0.048
PD1671	-	Two-component, response regulator	-0.952	0.002
PD1013	-	Uncharacterized protein	-0.952	0.012
PD1967	*ppx*	Exopolyphosphatase	-0.957	0.002
PD1789	-	Uncharacterized protein	-0.958	0.001
PD0772	-	Phage-related protein	-0.963	0.020
PD0368	*higA*	Proteic killer suppression protein	-0.966	0.002
PD1620	-	Outer membrane protein assembly factor BamB	-0.968	0.000
PD0008	-	Uncharacterized protein	-0.968	0.001
PD0864	*xthA*	Exodeoxyribonuclease III	-0.982	0.000
PD1597	-	Uncharacterized protein	-0.986	0.014
PD1655	*ttg2B*	Toluene tolerance protein	-0.988	0.000
PD1592	-	Uncharacterized protein	-0.991	0.043
PD0350	-	Succinate dehydrogenase membrane anchor subunit	-0.996	0.002
PD1743	*nusB*	N utilization substance protein B homolog (Protein NusB)	-1.003	0.001
PD2002	*rplL*	50S ribosomal protein L7/L12	-1.004	0.001
PD1284	*algU*	RNA polymerase sigma-H factor	-1.006	0.000
PD1599	-	Phage-related protein	-1.018	0.010
PD0747	*oxyR*	Oxidative stress transcriptional regulator	-1.025	0.000
PD0084	*rplS*	50S ribosomal protein L19	-1.035	0.003
PD1709	*mopB*	Outer membrane protein	-1.042	0.000
PD0312	-	Uncharacterized protein	-1.046	0.001

PD0127	-	Uncharacterized protein	-1.054	0.000
PD0507	-	Pathogenicity protein	-1.058	0.000
PD0141	*fabG*	3-oxoacyl-[ACP] reductase	-1.058	0.001
PD1581	-	Uncharacterized protein	-1.061	0.000
PD0195	*rbfA*	Ribosome-binding factor A	-1.065	0.000
PD1510	-	Uncharacterized protein	-1.066	0.035
PD1945	*rpsF*	30S ribosomal protein S6	-1.069	0.002
PD0503	-	Uncharacterized protein	-1.083	0.000
PD1411	*aspC*	Aminotransferase	-1.1	0.000
PD0497	-	Uncharacterized protein	-1.107	0.002
PD2108	-	Uncharacterized protein	-1.112	0.003
PD1547	-	Uncharacterized protein	-1.113	0.000
PD1700	-	Uncharacterized protein	-1.133	0.022
PD2085	-	Uncharacterized protein	-1.138	0.020
PD2018	*ipk*	4-diphosphocytidyl-2-C-methyl-D-erythritol kinase (CMK)	-1.17	0.000
PD0743	-	Uncharacterized protein	-1.172	0.016
PD2003	*rplJ*	50S ribosomal protein L10	-1.175	0.001
PD0400	*greA*	Transcription elongation factor GreA (Transcript cleavage factor GreA)	-1.185	0.001
PD2025	-	Uncharacterized protein	-1.196	0.006
PD0488	*rpmB*	50S ribosomal protein L28	-1.197	0.001
PD1111	-	Uncharacterized protein	-1.2	0.001
PD1993	-	Temperature acclimation protein B	-1.201	0.001
PD0811	*fis*	Putative Fis-like DNA-binding protein	-1.215	0.002
PD0546	*ispF*	2-C-methyl-D-erythritol 2,4-cyclodiphosphate synthase (MECDP-synthase)	-1.216	0.005
PD1710	*dsbB*	Disulfide bond formation protein B (Disulfide oxidoreductase)	-1.228	0.000
PD1094	*FI*	Phage-related contractile tail sheath protein	-1.237	0.000
PD0824	*hsf*	Surface protein	-1.272	0.000
PD0003	*recF*	DNA replication and repair protein RecF	-1.272	0.000
PD2122	*rnpA*	Ribonuclease P protein component (RNase P protein)	-1.279	0.001
PD1345	-	Uncharacterized protein PD_1345	-1.279	0.001
PD0791	-	Uncharacterized protein	-1.284	0.001
PD1050	-	Uncharacterized protein	-1.305	0.001
PD1285	-	Uncharacterized protein	-1.32	0.000
PD0528	-	Uncharacterized protein	-1.323	0.000
PD1442	*rpmA*	50S ribosomal protein L27	-1.335	0.000
PD1752	*yojH*	Probable malate:quinone oxidoreductase (EC 1.1.5.4)	-1.345	0.001
PD1112	-	Uncharacterized protein	-1.354	0.003
PD0666	*infA*	Translation initiation factor IF-1	-1.357	0.000
PD0244	-	Uncharacterized protein	-1.381	0.022
PD0489	*rpmG*	50S ribosomal protein L33	-1.386	0.000
PD0577	*dcm*	Site-specific DNA-methyltransferase	-1.39	0.000
PD1960	*rpsB*	30S ribosomal protein S2	-1.447	0.000
PD0657	-	Uncharacterized protein	-1.453	0.006

PD2005	*rplK*	50S ribosomal protein L11	-1.474	0.000
PD2117	-	Uncharacterized protein	-1.505	0.000
PD1732	-	Frame shift	-1.517	0.000
PD0498	-	Uncharacterized protein	-1.543	0.000
PD0217	-	Uncharacterized protein	-1.637	0.000
PD0918	-	Phage-related protein	-1.671	0.000
PD0298	-	Uncharacterized protein	-1.681	0.000
PD1113	-	Lysozyme (EC 3.2.1.17)	-1.74	0.000
PD0299	-	Uncharacterized protein	-1.743	0.011
PD1507	-	Uncharacterized protein	-1.781	0.000
PD1130	-	Uncharacterized protein	-1.869	0.000
PD0243	-	Uncharacterized protein	-1.89	0.001
PD0556	-	Uncharacterized protein	-1.929	0.000
PD1723	-	Uncharacterized protein	-1.97	0.000
PD1786	-	Uncharacterized protein	-2.226	0.001
PD0216	-	Colicin V	-3.079	0.000
PD1790	-	Uncharacterized protein	-3.116	0.000
PD1121	-	Uncharacterized protein	-3.432	0.000
PD1245	-	Uncharacterized protein	-4.826	0.000
PD0921	-	Uncharacterized protein	-4.955	0.000
PD0919	-	Uncharacterized protein	-5.022	0.000
PD0920	-	Uncharacterized protein	-5.087	0.000

Table S2. Differentially expressed secreted proteins in the *prtA* mutant.

**Description**	**Gene name**	**FC (*prtA*/ WT)**	**p-value**
Argininosuccinate synthase argG	argG	3.007	0.006
Polyphosphate-selective porin	oprO	2.62	0.000
30S ribosomal protein S5	rpsE	2.296	0.028
Uncharacterized protein	PD0955	1.976	0.033
Fimbrial protein	pilA	1.974	0.018
50S ribosomal protein	rplJ	1.955	0.035
Outer membrane protein XadA	xadA	1.871	0.000
Uncharacterized protein	PD1684	1.82	0.012
50S ribosomal protein L3	rplC	1.792	0.036
50S ribosomal protein L11	rplK	1.738	0.037
DNA-directed RNA polymerase subunit alpha	rpoA	1.693	0.045
50S ribosomal protein L1	rplA	1.683	0.033
DNA-directed RNA polymerase subunit beta'	rpoC	1.676	0.017
Transcription termination factor Rho	rho	1.597	0.033
Rod shape-determining protein	mreB	1.58	0.028
Ketol-acid reductoisomerase	ilvC	1.579	0.014
GTP-binding elongation factor protein	typA	1.569	0.037

ATP synthase subunit alpha	atpA	1.549	0.037
ATP synthase subunit b	atpF	1.547	0.015
50S ribosomal protein L30	rpmD	1.448	0.039
ATP-dependent RNA helicase DeaD	deaD	1.42	0.029
DNA-directed RNA polymerase subunit beta	rpoB	1.37	0.042
Succinate dehydrogenase flavoprotein subunit	sdhA	1.308	0.037
Transcription termination/antitermination protein NusA	nusA	1.303	0.035
Bacteriocin	frpC	0.757	0.037
60 kDa chaperonin	groL	0.754	0.021
Metallopeptidase	PD1570	0.751	0.037
6-phosphogluconate dehydratase	edd	0.721	0.015
Glycine dehydrogenase (decarboxylating)	gcvP	0.713	0.027
Serine protease pspB	pspB	0.71	0.036
Polyribonucleotide nucleotidyltransferase	pnp	0.707	0.005
OmpA family protein	ompA	0.696	0.000
Oxidoreductase	PD2039	0.696	0.037
10 kDa chaperonin	groS	0.691	0.029
N-acetylornithine carbamoyltransferase	argF'	0.686	0.029
Phosphoglucomutase	xanA	0.675	0.000
D-3-phosphoglycerate dehydrogenase	serA	0.665	0.008
Periplasmic protease	mucD	0.663	0.017
Chaperone protein ClpB	clpB	0.662	0.010
RNA polymerase-binding transcription factor DksA	dksA	0.658	0.007
Isocitrate dehydrogenase	icd	0.657	0.001
Bifunctional purine biosynthesis protein PurH	purH	0.654	0.005
Chaperone protein	PD0061	0.651	0.049
Outer membrane protein P6	PD0895	0.647	0.027
Uncharacterized protein	PD0121	0.638	0.003
Thiol:disulfide interchange protein	dsbA	0.634	0.023
Isocitrate/isopropylmalate dehydrogenase	icdA	0.633	0.001
Oligopeptidase A	prlC	0.63	0.001
Subunit C of alkyl hydroperoxide reductase	ahpC	0.63	0.003
ABC transporter sulfate binding protein	sbp	0.625	0.008
Inorganic pyrophosphatase	ppa	0.625	0.006
Uncharacterized protein	PD0896	0.624	0.020
Citrate synthase	gltA	0.623	0.039
Valine--tRNA ligase	valS	0.615	0.001
3-hydroxydecanoyl-[acyl-carrier-protein] dehydratase	fabA	0.614	0.037
2,3,4,5-tetrahydropyridine-2,6-dicarboxylate N-succinyltransferase	dapD	0.611	0.000
Dihydroorotase	pyrC	0.611	0.005
Probable cytosol aminopeptidase	pepA	0.608	0.000
Ribosome-recycling factor	frr	0.602	0.014
Uncharacterized protein	PD0904	0.593	0.000
Uncharacterized protein	PD1556	0.589	0.040
Glyceraldehyde-3-phosphate dehydrogenase	gapA	0.588	0.001
50S ribosomal protein L9	rplI	0.584	0.000

Asparagine--tRNA ligase	asnS	0.584	0.005
Zinc protease	PD1853	0.581	0.000
S-adenosylmethionine synthase	metK	0.574	0.003
Aconitase	rpfA	0.561	0.000
Peptidase (M20/M25/M40 family)	PD1850	0.561	0.000
Phosphoserine aminotransferase	serC	0.561	0.037
Alanyl dipeptidyl peptidase	PD1300	0.557	0.000
Peptidyl-dipeptidase	dcp	0.551	0.000
Dipeptidyl aminopeptidase	ptrB	0.546	0.000
Peptidyl-prolyl cis-trans isomerase	PD1168	0.544	0.000
Thioredoxin	yneN	0.544	0.024
Toluene tolerance protein	ttg2D	0.544	0.000
Chaperone protein HtpG	htpG	0.542	0.000
Malate dehydrogenase	mdh	0.51	0.000
Electron transfer flavoprotein alpha subunit	etfA	0.505	0.000
Uncharacterized protein	PD0657	0.502	0.001
Cysteine synthase	cysK	0.498	0.019
Organic hydroperoxide resistance protein	ohr	0.474	0.000
Superoxide dismutase	sodA	0.468	0.000
2-dehydro-3-deoxyphosphooctonate aldolase	kdsA	0.46	0.015
Chaperone SurA	surA	0.452	0.000
Uncharacterized protein	PD1786	0.436	0.000
Probable S-methyl-5'-thioinosine phosphorylase	mtnP	0.422	0.004
Uncharacterized protein	PD0090	0.403	0.039
Chaperone protein DnaK	dnaK	0.401	0.000
Uncharacterized protein	PD0858	0.397	0.000
Uncharacterized protein	PD1028	0.389	0.000
Superoxide dismutase	sodM	0.375	0.002
Uncharacterized protein	PD0956	0.347	0.000

Table S3. Enrichment analysis of gene ontology terms from differentially expressed transcripts in the *prtA* mutant.

Up-regulated				
**ID**	**Description**	***P***	***G****	***G*90% CI**
GO:0003677	DNA binding	0	0.39	[0.32;0.47]
GO:0008235	metalloexopeptidase activity	0	0.84	[0.84;0.84]
GO:0006561	proline biosynthesis	0.01	0.84	[0.84;0.84]
GO:0009245	lipid A biosynthesis	0.01	0.55	[0.55;0.55]
GO:0006760	folic acid and derivative metabolism	0.01	0.89	[0.89;0.89]
GO:0030163	protein catabolism	0.01	1.00	[1.00;1.00]
GO:0045449	regulation of transcription	0.02	0.59	[0.44;0.73]
GO:0006865	amino acid transport	0.03	0.70	[0.70;0.70]
GO:0009432	SOS response	0.03	0.48	[0.49;0.49]
GO:0045892	negative regulation of transcription	0.03	0.84	[0.84;0.84]
GO:0004239	methionyl aminopeptidase activity	0.03	1.00	[1.00;1.00]
GO:0006974	response to DNA damage stimulus	0.04	0.35	[0.27;0.48]
GO:0015995	porphyrin biosynthesis	0.04	0.59	[0.59;0.59]
GO:0042626	ATPase activity, coupled to transmembrane movement	0.04	0.62	[0.63;0.63]
GO:0015628	type II protein secretion system	0.04	0.62	[0.48;0.76]
GO:0009405	pathogenesis	0.04	0.59	[0.59;0.59]
GO:0000155	two-component sensor molecule activity	0.05	0.45	[0.45;0.45]
Down-regulated				
**ID**	**Description**	***P***	***G****	***G*90% CI**
GO:0003723	RNA binding	0	0.43	[0.40;0.46]
GO:0006412	protein biosynthesis	0	0.58	[0.56;0.61]
GO:0003735	structural constituent of ribosome	0	0.67	[0.67;0.67]
GO:0005840	ribosome	0	0.67	[0.67;0.67]
GO:0030529	ribonucleoprotein complex	0	0.65	[0.65;0.65]
GO:0019843	rRNA binding	0	0.61	[0.61;0.61]
GO:0006099	tricarboxylic acid cycle	0	0.68	[0.68;0.68]
GO:0006541	glutamine metabolism	0	0.67	[0.67;0.67]
GO:0004088	carbamoyl-phosphate synthase activity	0	1.00	[1.00;1.00]
GO:0015935	small ribosomal subunit	0.01	0.71	[0.71;0.71]
GO:0009165	nucleotide biosynthesis	0.01	0.71	[0.71;0.71]
GO:0009089	lysine biosynthesis via diaminopimelate	0.01	0.80	[0.80;0.80]
GO:0003989	acetyl-CoA carboxylase activity	0.01	0.84	[0.84;0.84]
GO:0006353	transcription termination	0.02	0.84	[0.84;0.84]
GO:0015450	protein translocase activity	0.02	0.71	[0.71;0.71]
GO:0016874	ligase activity	0.02	0.28	[0.23;0.37]
GO:0004289	subtilase activity	0.02	0.84	[0.84;0.84]
GO:0004834	tryptophan synthase activity	0.02	1.00	[1.00;1.00]
GO:0006098	pentose-phosphate shunt	0.02	1.00	[1.00;1.00]
GO:0000049	tRNA binding	0.03	0.65	[0.65;0.65]
GO:0042254	ribosome biogenesis and assembly	0.03	1.00	[1.00;1.00]
GO:0016868	intramolecular transferase activity, phosphotransferases	0.03	0.71	[0.71;0.71]
GO:0009317	acetyl-CoA carboxylase complex	0.03	0.84	[0.84;0.84]
GO:0006568	tryptophan metabolism	0.03	0.71	[0.71;0.71]
GO:0004086	carbamoyl-phosphate synthase activity	0.03	1.00	[1.00;1.00]

GO:0016829	lyase activity	0.04	0.28	[0.24;0.33]
GO:0016114	terpenoid biosynthesis	0.04	0.63	[0.63;0.63]
GO:0009306	protein secretion	0.04	0.60	[0.55;0.66]
GO:0016616	oxidoreductase activity, acting on the CH-OH group of donors	0.04	0.71	[0.59;0.80]
GO:0000104	succinate dehydrogenase activity	0.04	1.00	[1.00;1.00]
GO:0009231	riboflavin biosynthesis	0.05	0.80	[0.71;0.85]
GO:0006096	glycolysis	0.05	0.46	[0.46;0.46]

* G is the gamma measure of statistical association and G90% is its 90% credibility interval ("error-bar").

Complete method for Western blot with anti-PrtA

To detect PrtA in *X. fastidiosa*, a polyclonal antibody was generated in rabbit by injecting synthetic peptides corresponding to structural epitopes (GenScript, NJ). Anti-PrtA was diluted in PBS-M 1% (PBS plus 1% non-fat dried milk) at a dilution of 1:500. Polyclonal anti-PrtA was HRP-conjugated. Blocking and washing used PBS-M 5% (PBS plus 5% non-fat dried milk) and PBS-T 0.1% (PBS plus 0.1% Tween 20), respectively, and the blot was developed using ECL Plus Reagents (GE Life Sciences, USA). The blot was visualized using a ChemiDoc-It TS2 (BioRad, CA) imaging instrument.

Complete method for protein extraction and proteomic data analysis

Six culture replicates were grown in PD3 media for seven days (120 rpm at 28ºC) and cells were separated by centrifugation at 20,000 g. The supernatant was concentrated using Amicon Ultra 15-Centrifigual Filter units (Millipore, Ma) with a three kDa membrane. Total protein was extracted from the resulting fraction using a ProteoExtract Protein Precipitation Kit (Millipore, MA). The resulting protein pellet was solubilized in 400 μL 50 mM triethyl ammonium bicarbonate (TEAB) and 100 μL was taken for trypic digestion. 500 mM tris(2-carboxyethyl)-phosphine (TCEP) (Pierce, Rockford, IL) was added to a final

concentration of 10 mM and samples were incubated 10 min at 90ºC to reduce disulfide bonds. Next, 110 mM iodoacetamide (IAA) was added to a final concentration of 15 mM and incubated one hour at room temperature, followed by the addition of 20 µL DTT to quench the IAA reaction. A 1:25 (enzyme:protein) ratio of trypsin (Promega) was added to 100 µg of the protein sample and incubated at 37ºC overnight. Subsequently, samples were desalted using C18 Macro Spin columns (Nest Group) and dried by vacuum centrifugation. The desalted and lyophilized samples were resuspended in 50 mM TEAB and ~30 ug of tryptic-digested peptides were taken for TMT labeling. TMT labeling was performed on each aliquot with reporter ions m/z = 126.1, 127.1, 128.1, and 129.1 in 41 µL ethanol and aliquots were incubated 60 min at room temperature. Eight µL hydroxylamine 5% (v:v) was added to quench the reaction and samples were vacuum-centrifuged prior to desalting using C18 Macro Spin columns (Nest Group, MA). Samples were vacuum-centrifuged once more prior to strong cation exchange (SCX) fraction.

Strong cation exchange (SCX) was carried out using the SCX SpinTips Sample Prep Kit (ProteaBio, WV). Each aliquot was resuspended in 50 µL of designated buffer and ~10 µg of each sample was pooled prior to SCX fractionation. Samples were fractionated by stepwise addition of 20, 40, 60, 80, 100, 150, 250, and 500 mM ammonium formate in 10% acetonitrile. All eight fractions, including the initial binding flow through, were vacuum-centrifuged to remove any acetonitrile and then desalted using C18 Macro Spin columns (Nest Group, MA). LC separation was carried out on a Waters Nano Acquity UHPLC (Waters Corporation, MA) with a Proxeon nanospray source. Each SCX fraction (nine total) was reconstituted in 2% acetonitrile/ 0.1% trifluoroacetic acid and 1 µg loaded onto a 100 micron x 25 mm Magic C18 100Å 5U reverse phase trap, where they were desalted online before separation on a 75 micron x 150 mm Magic C18 200Å 3U reverse phase column.

Peptides were eluted using a gradient of 0.1% formic acid (A) and 100% acetonitrile (B) with a flow rate of 300 nL/min. A 60 min gradient was run with 5% to 35% B over 50 min, 35% to 80% B over three min, 80% B for one min, 80% to 5% B over one min, and finally held at 5% B for five min.

Mass spectra were collected on an Orbitrap Q Exactive Plus mass spectrometer (Thermo Fisher Scientific, CA) in a data-dependent mode with one MS precursor scan followed by 15 MS/MS scans. All MS/MS samples were analyzed using X! Tandem (The GPM, thegpm.org; version X! Tandem Sledgehammer (2013.09.01.1)). X! Tandem was set up to search the Uniprot Glycine max database (64636 entries, March 2014), the *X. fastidiosa* (strain Temecula1/ATCC 700964) database (2008 entries, March 2014), the cRAP database of common laboratory contaminants (http://www.thegpm.org/crap/) and an equal number of reverse decoy sequences, assuming the digestion enzyme trypsin. X! Tandem was searched with a fragment ion mass tolerance of 20 ppm and a parent ion tolerance of 20 ppm. TMT6plex of lysine and the N-terminus was specified in X! Tandem as a fixed modification. Glu->pyro-Glu of the N-terminus, ammonia-loss of the N-terminus, gln->pyro-Glu of the N-terminus, deamidated of asparagine and glutamine, oxidation of methionine and tryptophan, dioxidation of methionine and tryptophan, and acetyl of the n-terminus were specified in X! Tandem as variable modifications. Scaffold Q+ (version Scaffold_4.4.0, Proteome Software Inc., Portland, OR) was used to analyze peptide and protein identifications and obtain TMT reporter ion intensities from the MS/MS spectra. Peptide identifications were accepted if they could be established at a 99.0% probability by the Scaffold Local FDR algorithm, which corresponded to a 0.20% spectra decoy FDR and a 5.0% protein decoy FDR with one identified peptide per protein. Protein probabilities were assigned by the Protein Prophet algorithm[2]. Proteins that contained similar peptides and

that could not be differentiated based on MS/MS analysis alone were grouped to satisfy the principles of parsimony. Proteins sharing significant peptide evidence were grouped into clusters. Acquired intensities in the experiment were globally normalized across all acquisition runs. Individual quantitative samples were normalized within each acquisition run. Intensities for each peptide identified were normalized within the assigned protein. The reference channels were normalized to produce a 1:1 fold change. All normalization calculations were performed using medians to multiplicatively normalize data. Peptide-level intensities were log transformed and normalized using the back-fitting approach as described[3], with the exception that they were not normalized for label effects since label was confounded with a strain. Tests for differential protein expression were conducted using linear mixed effects models with a fixed effect for strain and random effects for peptide and sample. P-values for the strain effect were FDR-adjusted. Analyses were conducted using the R, version 3.1.0 (http://www.R-project.org/). Mixed effects modeling was conducted using the R package nlme, version 3.1-117[4].

Complete method for RNA extraction and RNA-seq library preparation

Bacterial strains were grown in six replicates for eight days in 50 mL PD3 media at 28ºC with 120 rpm constant shaking. After obtaining a pellet by centrifuging at 10,000 g for 1 min, RNA was extracted using the MasterPure Complete DNA and RNA Purification Kit from Epicenter (Madison, WI) following manufacturer's directions. Next, ribosomal RNA was depleted using the Ribo-Zero rRNA Removal Kit for Gram-negative bacteria epicenter (Madison, WI) exactly as described by the manufacturer. Strand-specific RNA-seq libraries were generated by the UC Davis Genome Center DNA Technologies Core Facility from the ribo-depleted RNA samples using an Apollo 324 liquid handler (Wafergen, CA) and PrepX

RNA library preparation kits (Wafergen) following the instructions of the manufacturer. After a cleanup step using 1x volume of Ampure XP beads (Beckman Coulter, CA), the RNA-seq libraries were PCR-amplified using Phusion High-Fidelity polymerase (NEB, MA) following standard procedures, cleaned up again using a 1x volume of Ampure XP beads, and then quantified by fluorometry (Qubit; LifeTechnologies, CA). Libraries were analyzed with a Bioanalyzer 2100 instrument (Agilent, CA) and then pooled in equimolar ratios according to the fluorometric measurements. The pooled library was quantified by qPCR with a Kapa Library Quant kit (Kapa, South Africa) and sequenced on one lane of an Illumina HighSeq 2500 (Illumina, CA).

Transcriptome data analysis

Bioinformatics data analysis was performed by Monica Britton of the UC Davis Genome Center Bioinformatics Core Facility. Read quality and contamination were assessed with FastQC v.0-.10.1 (http://www.bioinformatics.babraham.ac.uk/projects/fastqc/). Scythe v.0.991 and Sickle v.1.210 (https://github.com/ucdavis-bioinformatics) were used for Illumina adapter and quality-based trimming, respectively. Reads trimmed to less than 25 bases were discarded. Genome and annotation data used in this analysis can be found at ftp://ftp.ncbi.nih.gov/genomes/Bacteria/Xylella_fastidiosa_Temecula1_uid57869/. Reads were aligned to the Temecula 1 genome using bowtie2 v.2.1.0[5]. These alignments showed a high level of transcription in a previously unannotated region (genomic coordinates 989100-989300). A reference transcriptome was generated from the NCBI files, using the gffread program within cufflinks v.2.1.1[6]. This transcriptome was augmented with the sequences of rRNA, the highly expressed unannotated region ("Tem1_989300_989100_RC"), and the transgenes (Kan_R, PrtA_GentR) used in this

research. BWA's short read aligner v.0.6.2[7] was then used to align the reads to the augmented transcriptome. Raw counts per gene were generated from the bwa alignments using sam2counts.py (https://github.com/ucdavis-bioinformatics). The raw counts were statistically analyzed with edgeR[8] to produce tables of expression values and fold changes and diagnostic plots.

Enrichment of gene ontology terms of differentially expressed transcripts was performed with BayGO[9] using 500 simulations and a P-value cutoff of 0.05.

References

1 Matsumoto, A., Young, G. M. & Igo, M. M. Chromosome-based genetic complementation system for Xylella fastidiosa. *Appl Environ Microb* **75**, 1679-1687, doi:10.1128/AEM.00024-09 (2009).

2 Nesvizhskii, A. I., Keller, A., Kolker, E. & Aebersold, R. A statistical model for identifying proteins by tandem mass spectrometry. *Analytical chemistry* **75**, 4646-4658 (2003).

3 Oberg, A. L. *et al.* Statistical analysis of relative labeled mass spectrometry data from complex samples using ANOVA. *J Proteome Res* **7**, 225-233, doi:Doi 10.1021/Pr700734f (2008).

4 Pinheiro J, B. D., DebRoy S, Sarkar D and R Core Team. nlme: Linear and Nonlinear Mixed Effects Models. *R package version 3.1-118* (2014).

5 Langmead, B. & Salzberg, S. L. Fast gapped-read alignment with Bowtie 2. *Nature methods* **9**, 357-359, doi:10.1038/nmeth.1923 (2012).

6 Roberts, A., Pimentel, H., Trapnell, C. & Pachter, L. Identification of novel transcripts in annotated genomes using RNA-Seq. *Bioinformatics* **27**, 2325-2329, doi:Doi 10.1093/Bioinformatics/Btr355 (2011).

7 Li, H. & Durbin, R. Fast and accurate short read alignment with Burrows-Wheeler transform. *Bioinformatics* **25**, 1754-1760, doi:10.1093/bioinformatics/btp324 (2009).

8 Robinson, M. D., McCarthy, D. J. & Smyth, G. K. edgeR: a Bioconductor package for differential expression analysis of digital gene expression data. *Bioinformatics* **26**, 139-140, doi:10.1093/bioinformatics/btp616 (2010).

9 Vencio, R. Z., Koide, T., Gomes, S. L. & Pereira, C. A. BayGO: Bayesian analysis of ontology term enrichment in microarray data. *BMC Bioinformatics* **7**, 86, doi:10.1186/1471-2105-7-86 (2006).
